# Improvement of hybrid polyvinyl chloride/dapsone membrane using synthesized silver nanoparticles for the efficient removal of heavy metals, microorganisms, and phosphate and nitrate compounds from polluted water†

**DOI:** 10.1039/d4ra03810j

**Published:** 2024-06-18

**Authors:** Hesham Moustafa, Mohamed A. Shemis, Emad M. Ahmed, Heba Isawi

**Affiliations:** a Polymer Metrology & Technology Department, National Institute of Standards (NIS) Tersa Street, El Haram, P.O. Box 136 Giza 12211 Egypt hesham.moustafa21@gmail.com +20 2338 6745 1 +20 0173 4580 0; b Bioanalysis Laboratory, National Institute of Standards (NIS) Tersa Street, El Haram, P.O. Box 136 Giza 12211 Egypt; c Department of Biochemistry and Molecular Biology, Theodore Bilharz Research Institute Giza Egypt; d Department of Physics, College of Science, Taif University Taif Saudi Arabia; e Water Treatment and Desalination Unit, Hydrogeochemistry Department, Water Resources and Desert Soils Division, Desert Research Center P.O.B. 11753 Cairo Egypt

## Abstract

Heavy metals exist in different water resources and can threaten human health, inducing several chronic illnesses such as cancer and renal diseases. Therefore, this work dealt with the fabrication of highly efficient nanomembranes based on silver nanoparticle (Ag NP)-doped hybrid polyvinyl chloride (PVC) by dapsone (DAP) using an *in situ* method. Fourier-transform infrared (FT-IR) spectroscopy and X-ray diffraction (XRD) analysis were used to confirm the hybridization of PVC as well as the crystalline structure of hybrid PVC nanocomposites. Three varying proportions of Ag NPs (*i.e.*, 0.1, 0.2, and 0.3%) were used to fabricate hybrid PVC-DAP nanomembranes. The Brunauer–Emmet–Teller (BET) method was used to estimate membrane surface area, porosity and distribution of pore volume. The mechanical strength and antibacterial properties of the cased films notably improved when Ag NPs were added depending on the NP ratio inside the matrix. Results obtained from adsorption experiments of PVC-DAP nanomembranes at 35 °C revealed that the optimum nanomembrane was achieved at 0.2% NPs and its percentage of removal effectiveness ranged from 71 to 95% depending on the ion type. The surface morphology of the PVC-DAP-0.2 Ag NPs before and after the adsorption process of the metal ions was analyzed using SEM-EDX. Moreover, the impact of other parameters such as the initial concentrations, pH media, temperature, and contacting time, on the adsorption efficiency of PVC-DAP-0.2 Ag NPs was also investigated. Furthermore, kinetic and adsorption isotherm models were suggested to describe the adsorption efficiency of the PVC-DAP-0.2 Ag NP membrane, and the uptake mechanism of metal ion removal was studied. The obtained outcomes for these fabricated nanomembranes demonstrated that they could be potential candidates for water purification and other potential purposes including biomedical areas.

## Abstract 2

Highly efficient nanomembranes from hybrid PVC-DAP reinforced with Ag NPs for use in water purification and dialysis devices in the medical field.

## Graphical abstract

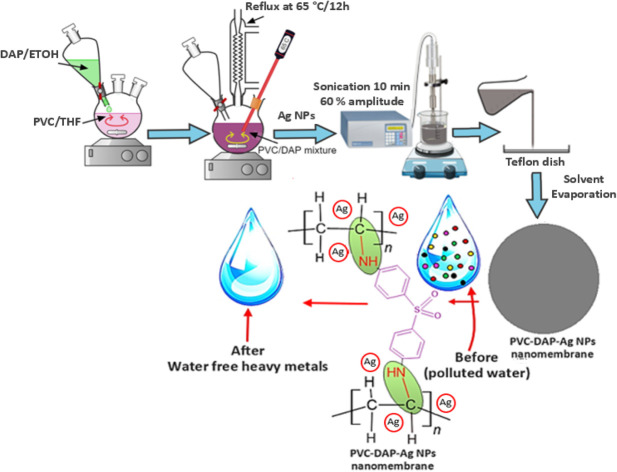

## 1. Introduction

For the time being, the consumption of water resources is continuously increasing, and the water contamination issue is worsening.^1^ Thus, chronic diseases based on polluted water by heavy metals are considered a major threat to human health and the environment due to the augmenting morbidity rate of such diseases globally. Among these diseases, cancer and chronic renal illnesses can harm human organs when these metals exceed their safe limits. Heavy metals exist in various sources including ground water resources and environmental pollutants, where the former is the main factor for irrigation worldwide. Therefore, heavy metals enter the human body through the food chain and polluted drinking water, causing many health risks even at very low concentrations because of their accumulation and nondegradability in the long term.^2–4^ Accordingly, prolonged body exposure to these metals when they exceed the safe limits induces severe diseases such as cancer, neurodegenerative disorders, and hemochromatosis (*i.e.*, iron overload), in addition to chronic renal illnesses.^5,6^ To tackle these shortcomings, nanomembrane-based smart polymeric matrices have been developed to enhance the removal of heavy metals from water resources and provide safe water owing to innovative nanomembranes for living on a healthy planet. Huge efforts^7–9^ have been made to explore these membranes as viable and cost-effective nanomembrane manufacturing. Regarding renal diseases, the kidney is considered the first attack organ by heavy metal toxicity that causes acute renal failure, leading mostly to death.

PVC is a synthetic plastic that is extensively utilized in various applications such as pipes, cable insulation, and medical apparatus because of its exceptional properties comparable with other synthetic thermoplastics.^10^ It possesses high strength and good physical and chemical properties, in addition to thermal stability.^11^ Furthermore, PVC is an inexpensive polymer with high resistance to chemicals, film forming ability, and good solubility in diverse organic solvents. In addition to its amazing PVC characteristics, it is an interesting polymer for membrane fabrication. Efforts^12–14^ have been made on PVC-based membranes for oily wastewater remediation. Besides PVC properties, Ghaedi *et al.*^15^ fabricated a PVC membrane as an optical sensor for detecting cupper ions (Cu^2+^) in different water resources. Aryanti *et al.*^16^ developed ultrafiltration nanomembrane-based PVC/polyethylene glycol reinforced with zinc oxide nanoparticles for river water remediation. Similarly, some studies^17–20^ have been conducted to blend or hybridize PVC with materials to achieve the processability and other properties of the PVC matrix. For instance, Fang *et al.*^21^ fabricated a positively charged nanomembrane from PVC-grafted-poly(*N*,*N*-dimethylaminoethyl methacrylate) using a crosslinking reaction for multivalent ion removal. However, Cai *et al.*^22^ prepared highly efficient separation membranes by incorporating inorganic nanomaterials inside the PVC matrix to enhance overall membrane efficiencies. Therefore, the grafting or hybridizing of PVC by these kinds of matters contributed to the rebirth of new alternatives for PVC matrices with sustainable properties. Therefore, (4,4 diaminodiphenyl sulfone) or dapsone (DAP) was utilized as a hybridizing material for the PVC matrix. Based on the DAP structure, it is considered a dual action as an antibiotic and anti-inflammatory therapy.^23,24^ A study by Williams *et al.*^25^ reported that DAP was the most widespread therapy for leprosy victims globally. Kawabata *et al.*^26^ studied the influence of ultraviolet (UV) lights and sunlight on the photodegradation of DAP in the aquatic medium. Moustafa *et al.*^24^ developed a decorated bioagent from DAP-capped TiO_2_ to boost the properties of the polyvinyl alcohol to be used in food-safe packaging and UV-shielding for biomedical purposes. Thus, the presence of primary amino groups in the DAP renders it highly reactive with several nanoparticles and polymer matrices to create new hybrid nanocomposites with amazing performance for end use materials.^27^ At present, Ag NPs have attracted great interest in the fabrication of antimicrobial and antifouling polymeric membranes.^8,28^ They are efficiently active in hindering the growth of a broad range of different microbes, including bacteria, fungi and viruses, through their interaction physically with microbe walls and media, resulting in cell death.^29,30^ Additionally, they can be used as reinforcing material inside polymeric matrices.^31^ A study by El Shehawy *et al.*^32^ reported that Ag NPs have the potential as bio-adsorbents to eliminate heavy metals from contaminated water. However, Ag NPs mostly tend to form agglomeration structures into the polymer matrix.^33^ To tackle the nanofiller agglomeration problem inside the matrix, the grafting of polymer or nanofiller treatment is necessary. To the best of our knowledge, no study has reported Ag NPs embedded in the hybridized PVC-DAP nanomembranes. Furthermore, sonication was employed in the nanomembrane preparation approach to achieve better homogeneity inside the membrane matrix. Consequently, this study focuses mainly on fabricating effective nanomembranes from Ag NPs doped inside hybrid PVC-DAP polymers to improve the removal of toxic metal ions and other biological impurities from water resources at an affordable cost.

In the present study, a facile hybridization of the PVC matrix using DAP material to obtain a hybrid PVC-DAP polymer was successfully performed. Varying ratios of Ag NPs were utilized as reinforcing agents in the PVC-DAP matrix to improve the nanomembrane properties and their long-term stability. The structure of hybrid PVC-DAP and the fabricated nanomembranes were investigated using FT-IR and XRD comparable with virgin PVC and pure DAP. The tensile, antibacterial and absorption efficiency properties of hybrid nanocomposites were metered. Moreover, the morphology of the optimized nanomembrane was assessed before and after the uptake of toxic metal ions through SEM-EDX visualization. The isotherm and kinetic models of adsorption removal efficiency and the uptake mechanism for PVC-DAP-0.2 Ag NPs were also discussed.

## 2. Materials and experimental techniques

### 2.1. Materials

Food-grade polyvinyl chloride (C_2_H_3_Cl, PVC, ACS grade) in a powder shape with a density of 1.40 g cm^−3^ was obtained from Sigma-Aldrich, Munich, Germany. Its average molecular weight was ∼43 000.^34^ The detailed synthesis of silver nanoparticles (Ag NPs) was previously reported in our article.^29^ The 4,4-diaminodiphenylsulfone or dapsone (C_12_H_12_N_2_O_2_S, DAP, ≥98%) was purchased from Alfa Aesar, USA. Anhydrous ferrous sulfate (FeSO_4_), manganese chloride (MnCl_2_), nickel chloride (NiCl_2_), lead acetate (Pb (CH_3_COO)_2_), disodium hydrogen phosphate heptahydrate (Na_2_HPO_4_·7H_2_O, MW = 268.07), uric acid (C_5_H_4_N_4_O_3_, MW = 168.11), and sodium nitrate (NaNO_3_, MW = 84.99) were used for analytical evaluation and were purchased from Sigma-Aldrich. All the chemicals and other reagents used in the tests were analytically pure and did not require any additional purification. A pH meter (3510, Jenway, UK) was used to adjust the pH using 0.1 M sodium hydroxide (NaOH, 98%) or hydrogen chloride (HCl, 30%) purchased from Sigma-Aldrich. The Inductively Coupled Argon Plasma-Mass Spectrometry (ICP-MS 6500 Duo (POEMS III)) by Thermo Jarrell Ash in the USA was used to determine the selected heavy metal ions ferric, manganese, nickel, and lead (Fe^3+^, Mn^2+^, Ni^2+^, and Pb^2+^). The phosphate (PO_4_^3−^) ions were determined calorimetrically through the phosphomolybdate method using a UV-visible spectrophotometer Thermo Unicam (Model 300, UK) at wavelength 700 or 880 nm, and NO_3_^−^ (nitrate) ions were determined at wavelength 430 nm. Uric acid was estimated as the total nitrogen by Kjeldahl steam distillation (Anonymous, 1994m).

### 2.2. Hybridizing PVC by dapsone

**Fig. 1:**
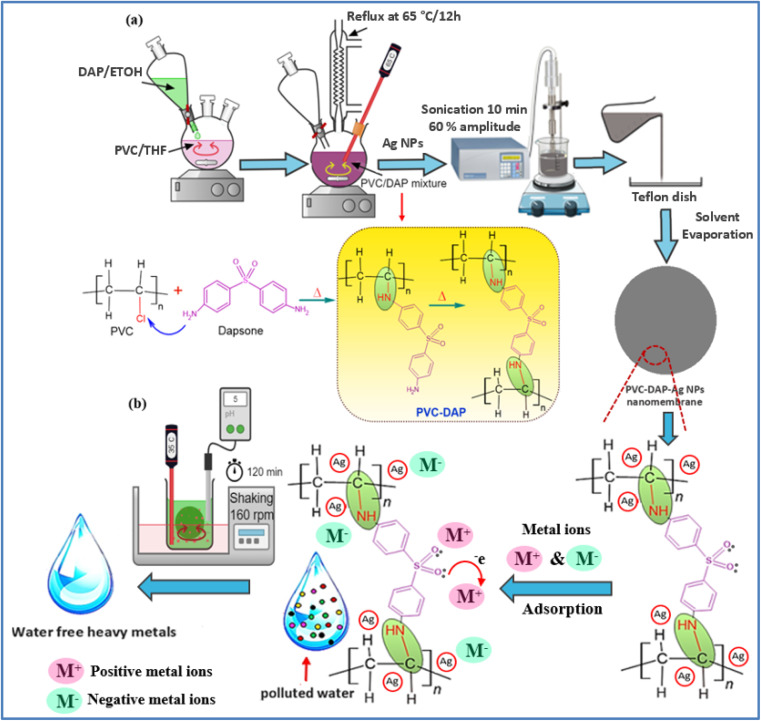
(a) Schematic representation showing the hybridization process of PVC by DAP to fabricate nanomembrane-based Ag NPs and (b) adsorption mechanism of heavy metal ions on the membrane surface.

Herein, hybridizing PVC by DAP was prepared by applying the *in situ* method as follows: 10 g of PVC was dissolved in a three-neck round flask containing 100 ml of THF under magnetic agitation until the complete dissolution of the polymer. Next, 3 g of DAP was dispersed in 50 ml ethanol and drop-wised into the polymer solution within approximately 30 min. Afterward, the mixture refluxed at 65 °C for 12 h with continuous agitation, as displayed in Fig. 1. Then, the mixture was cooled to ambient temperature and coagulated in distilled water, followed by triple rinsing with ethanol (70%) to eliminate the unreacted DAP and HCl as a byproduct. The obtained grafted PVC-DAP was dried in a laboratory oven at 70 °C for 6 h. After the drying stage, the nanomembrane-based hybrid PVC-DAP-doped Ag NPs were fabricated by dissolving 136 mg of PVC-DAP in 50 ml of THF with agitation for complete dissolution at 60 °C, then, three varying contents of Ag NPs (*i.e.*, 0.1, 0.2, and 0.5 wt%) were utilized and separately added to the dissolved polymer. The admixture was maintained under stirring for 15 min, followed by sonication for 10 min to fulfill the embedment of the Ag NPs and their dispersion inside the PVC-DAP matrix. The previous system was kept in an ice bath to avoid solvent evaporation. Thereafter, the obtained admixture was decanted in a 70 mm diameter glass Petri plate and left in the dark overnight under normal conditions to evaporate the solvent. The thickness of each cast film was adjusted by taking the same poured volume. After drying, the nanomembrane films were pulled off from the glass plates and kept in a laboratory desiccator for characterization and testing. The unfilled PVC and PVC-DAP matrices were also prepared, as mentioned above for comparison. The fabricated nanomembranes were coded based on the ratio of Ag NPs in the PVC-DAP matrix, such as PVC-DAP-0.1 Ag NPs, PVC-DAP-0.2 Ag NPs, and PVC-DAP-0.3 Ag NPs.

### 2.3. X-ray diffraction

The structure of pure DAP and hybrid unfilled polymer, as well as the morphology of absorbent films reinforced with Ag NPs, were metered using a Malvern Panalytical, 3rd generation Empyrean X-ray diffractometer equipped with Cu Kα radiation (45 kV, 40 mA, and *λ* = 0.15418 nm). The data were scanned over a 2*θ* range from 5 to 85° with a step size of 0.05 and at a counting time per step of 2 s per step for powder specimens, 0.013° for nanomembrane films, and at a sampling width of 0.010°.^35^ The crystallite size (*D*) of Ag NPs in terms of the peak width (*hkl*) for nanomembranes was determined using the Scherrer equation as follows:^29^
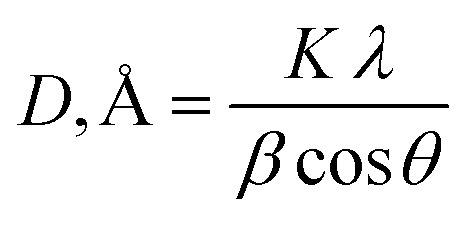
, *K* is a constant that is very close to unity, *λ* is the X-ray wavelength, *β* is the width at half maximum (FWHM), and *θ* is the diffraction angle. Additionally, hybridizing PVC by DAP and the filled nanocomposite films were verified using Bruker LUMOS II (Bruker, Hamburg, Germany). The FT-IR spectra were recorded with OPUS software, version 8.2, using the attenuated total reflection mode in the spectral range of 600–4000 cm^−1^ with a resolution of 4 cm^−1^ and 64 scans.^36^

### 2.4. SEM analysis

The surface morphology of the optimized nanomembrane before and after the metal ion adsorption process was investigated using scanning electron microscopy/Energy-dispersive X-ray spectroscopy (SEM Quanta FEG; EDX, Thermo scientific, dry/wet EDX). The fractured surfaces of the casted films were coated with a thin gold layer before the examination to avoid any electrostatic charging during observation.^37^

### 2.5. Mechanical properties

The tensile strength and elongation at break for casted absorbent films were conducted using a Zwick (Germany) universal tensile testing machine (Model Z010) equipped with a load cell of 100 N and a crosshead speed of 100 mm min^−1^, in accordance with ASTM D 882-18 standard. The dumbbell specimen's preparation and its environmental conditions were adjusted as described elsewhere.^36,38^ The mean value of five replicates for each film was recorded.

### 2.6. Brunauer–Emmet–Teller (BET)

An essential method for determining the specific surface area and pore size of PVC-DAP and PVC-DAP-0.2 Ag NPs using N_2_ adsorption–desorption techniques was Brunauer–Emmet–Teller (BET, Belsorp Minix, Microtrac, Japan).

### 2.7. Adsorption experiments

#### 2.7.1. Batch adsorption tests

One liter of ultrapure water was used to dissolve an appropriate amount of each anhydrous corresponding salt to create the standard solution (1000 mg L^−1^) of Fe^3+^, Mn^2+^, Ni^2+^, and Pb^2+^ ions (as mixed elements), while the standard solution (1000 mg L^−1^) of NO_3_^−^, PO_4_^3−^, and urea were prepared separately. To optimize the best sample available for element removal, about 0.1 g of each nanomembrane (virgin PVC, PVC-DAP), and their filled films (*i.e.*, PVC-DAP-0.1 Ag NPs, PVC-DAP-0.2 Ag NPs, and PVC-DAP-0.3 Ag NPs) was examined with 50 ml of 10 mg L^−1^ of element ion concentration at an appropriate pH for 120 min. When adjusting the adsorption conditions, the aqueous dilution of the stock solution created 50 ml of various element ion (5, 10, 15, 25, and 30 ppm) concentrations. Furthermore, the effects of changing the pH from 5 to 7.2 using 0.1 M NaOH and HCl solutions and contact times between 15 and 120 min were studied. Additionally, the impact of three temperatures (25, 35, and 45 °C) on the uptake of elemental ions was investigated. All the uptake tests were performed using an automated shaker at 160 rpm (Fig. 4(a)). When estimating the ability of element ions to be adsorbed, the variation in the concentrations of element ions in the solution before and after the uptake test and the weight of the optimal sample stated in mg g^−1^ were all considered. For the subsequent computations, each adsorption process received an average of three repetitions.

The following equations were used to compute the removal efficacy (*Q*%) and the adsorption capacity of the adsorbent, which covered the time interval from time *t* (*q*_*t*_) to stability time *q*_e_:1
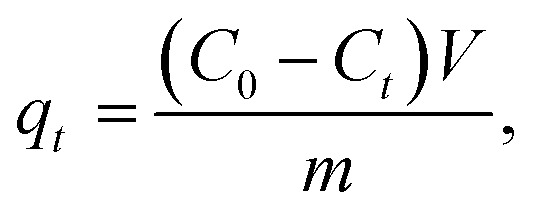
2
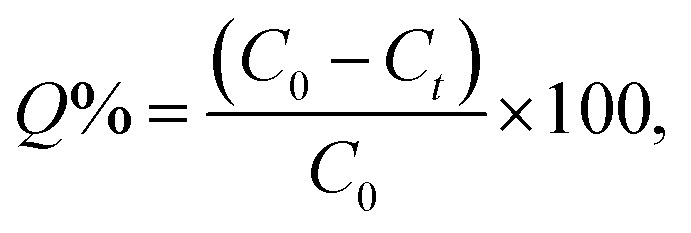
where *m* is the mass of the adsorbent (g), *V* is the volume of the adsorption material (adsorbate) solution (L), and *q*_*t*_ (mg g^−1^) is the quantity of element uptake on the adsorbent's unit mass at a time (*t*). The element ion concentrations *C*_0_ and *C*_t_ (mg L^−1^) are measured before and after uptake at time *t*, which ranges from 15 min to equilibrium time.

#### 2.7.2. Regeneration and reuse study

Through desorption testing, the mechanism of contaminant removal and the reusability of the element ions can be readily recognized. We investigated the leaching/desorption of several element ions from aqueous solutions using DI water and 0.1 M of both NaOH and HCl. A recyclable PVC-DAP-0.2 Ag NP membrane was generated by frequently washing it in DI water, which exposes it to four series of adsorption and desorption, and then constantly shaking it for 30 min at 25 °C at 160–170 rpm. The regeneration of Fe^3+^, Mn^2+^, Ni^2+^, Pb^2+^, NO_3_^−^, PO_4_^3−^, and urea into the PVC-DAP-0.2 Ag NP membrane to eliminate different contaminants was evaluated many times using batch adsorption/desorption tests with 50 ml of 10 mg L^−1^ of the selected element ions at an acceptable pH ranging from 5 to 7.2 under automated shaking at 160 rpm at room temperature. Before immersing the regenerating PVC-DAP-0.2 Ag NP membrane, the pH of the solution was adjusted. A similar process was used to evaluate the regenerated element ions and subsequent adsorption/desorption cycles. The regeneration efficacy (RE%) was calculated using the following equation:3
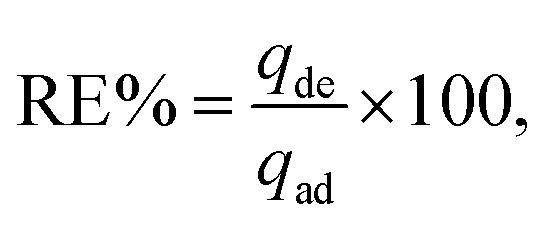
where *q*_de_ represents the quantity desorbed by each fluid and *q*_ad_ represents the amount adsorbed during loading.

#### 2.7.3. Application of the collected water samples using a PVC-DAP-0.2 Ag NP membrane

From the New Valley Governorate in Egypt, one groundwater specimen was taken to be tested using a PVC-DAP-0.2 Ag NP membrane. The residual solution was then retested after a 50 ml sample of the collected polluted samples was equilibrated with 0.15 g of the created PVC-DAP-0.2 Ag NP membrane for 120 min at 30 °C.

#### 2.7.4. Mathematical modeling

##### 2.7.4.1. Isotherm, kinetic studies, and thermodynamic parameters

Adsorption isotherms are used to represent the distribution of adsorbate molecules between the liquid and solid phases at the equilibrium of the adsorption process.^39^ The adsorption data were analyzed using four adsorption isotherms: the Langmuir, Freundlich, Temkin and Dubinin–Radushkevich (D–R) models (eqn (1)–(7) and Table S1†). To explore the adsorption process of the selected elements (Fe^3+^, Mn^2+^, Ni^2+^, Pb^2+^, NO_3_^−^, PO_4_^3−^, and urea) onto PVC-DAP-0.2 Ag NP membrane, two kinetic models (pseudo-first (PF) and second (PS) order kinetics) were carefully chosen (eqn (8) and (9) and Table S1†). The thermodynamic parameters for the selected element ion adsorption onto the PVC-DAP-0.2 Ag NP membrane were derived from the experimental data using Van't Hoff and Arrhenius equations (eqn (10) and (11) and Table S1†).

The Langmuir isotherm indicated a surface with uniform binding sites, equal sorption energies, monolayer adsorption, and no interactions between the adsorbed species. These were the most crucial presumptions for applying the Langmuir isotherm, such as the adsorbents (atoms, molecules, or ions) bonded to the active sites in precisely localized locations; only one substance was adsorbed at a time at each site, and each substance's energy level was constant across the entire surface regardless of what was adsorbed nearby.

One of the early correlations for irreversible and non-ideal adsorption was the Freundlich isotherm. Freundlich asserts that the adsorption region of an adsorbent surface is heterogeneous. This empirical relationship assumes that the heat and interest in adsorption were not spread uniformly across the adsorbent surface. Instead of single adsorption, this isotherm may be employed for multilayer adsorption. One of the key factors determining an adsorbent's efficacy is its adsorption kinetics, which characterizes the solute absorption rate by regulating the diffusion process and the residence duration of an adsorbate at the solid/solution interface. The pseudo-2nd-order kinetic model was concentrated on chemical adsorption, while the pseudo-1st-order kinetic model emphasized physical adsorption. The Temkin isotherm described the interactions between the compounds that were adsorbed. The values of each molecule's adsorption energy in an aqueous phase were considered when creating this isotherm model. This isotherm assumed that, disregarding the lower and upper concentration sets, the adsorption heat of all molecules in the layer decreases linearly with the active attachment sites as a function of temperature. The solute transfer in a solid–liquid sorption process is often characterized by either intraparticle diffusion, exterior mass transfer (boundary layer diffusion), or both. Adsorption at both the outer surface of the adsorbent and the transport of metal ion molecules from the bulk into its pores is possible. Either intraparticle diffusion (IPD) or film diffusion could be the adsorption rate-limiting stage.^40^ Adsorbates (metal ions) were carried to the adsorbent's exterior by film diffusion, whereas intraparticle diffusion occurred when adsorbates moved inside the adsorbent's pores. The slower of the two is the rate-determining step because they operate in succession. Using the following Weber–Morris equation (Table S1†), the potential for metal ion species to permeate into the inner spots of the adsorbent film (PVC-DAP-0.2 Ag NP membrane) was investigated:^41^ Table S1† depicts the IPD model expression and the intraparticle diffusion coefficient *K*_diff_.

To solve adsorption systems that highly produce rectangular isotherms and gain insight into the homogeneous energy distribution, the (D–R) adsorption isotherm empirical equation was specifically designed. The slope of the linearized isotherm equation, which provided information about the adsorption mechanism, was used to determine the adsorption energy. The model was typically used to distinguish between the mean free energy and the physical and chemical binding processes of the element cations. The effects of temperature on the adsorption of the chosen element onto the PVC-DAP-0.2 Ag NP membrane were investigated, and thermodynamic parameters (Δ*G*, Δ*H*, and Δ*S*) that described feasibility, spontaneity, and the type of adsorbate–adsorbent interactions were determined using the mathematical relationships shown in Table S1.†

### 2.8. Antibacterial assay

*Klebsiella pneumonia* (*K. pneumonia*) and *Staphylococcus aureus* (*S. aureus*) were chosen to represent G^−^ bacteria and G^+^ bacteria, respectively. The procedure for assessing antibacterial activity was conducted using the agar disc diffusion procedure, as reported elsewhere.^29,36^ At least duplicates were taken for each specimen.

## 3. Results and discussion

### 3.1. FT-IR analysis and X-ray diffraction

**Fig. 2:**
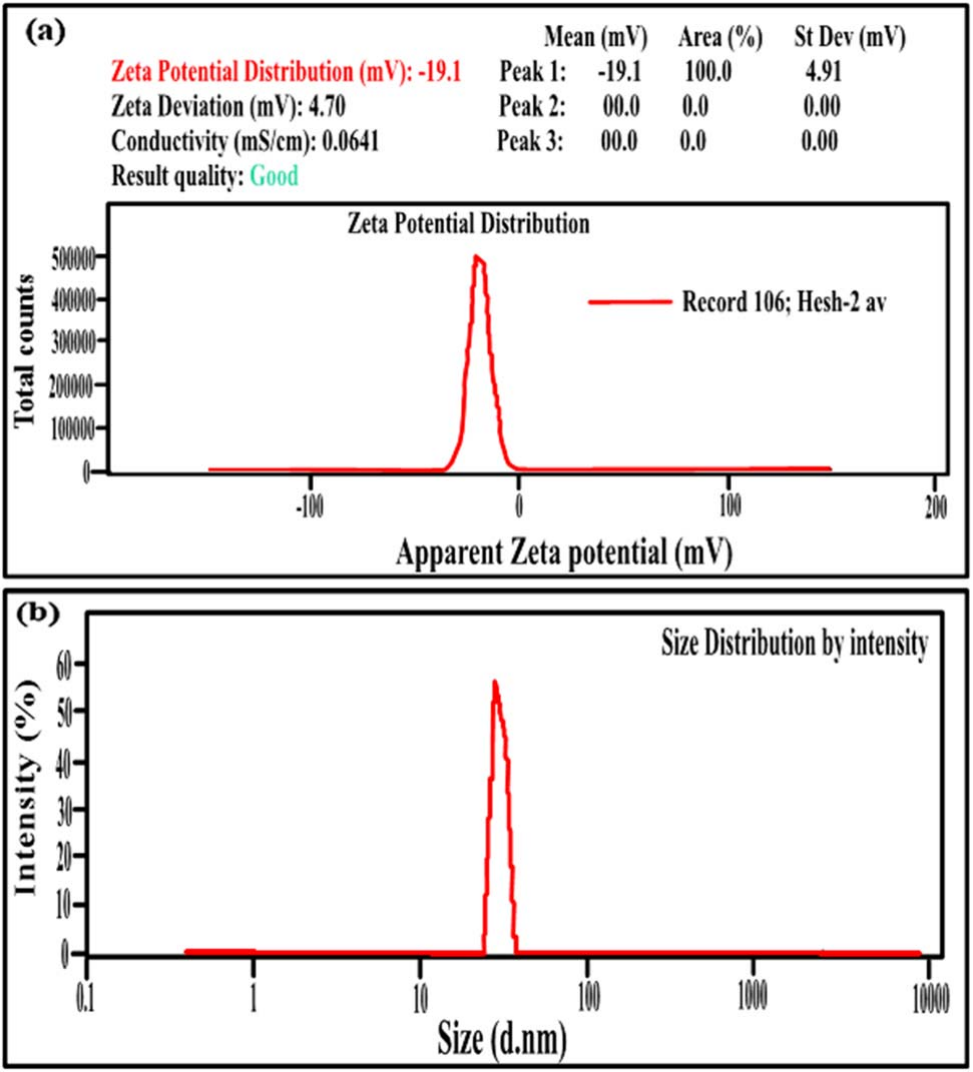
(a) Zeta potential of Ag NPs and their particle size distribution (b).

**Fig. 3:**
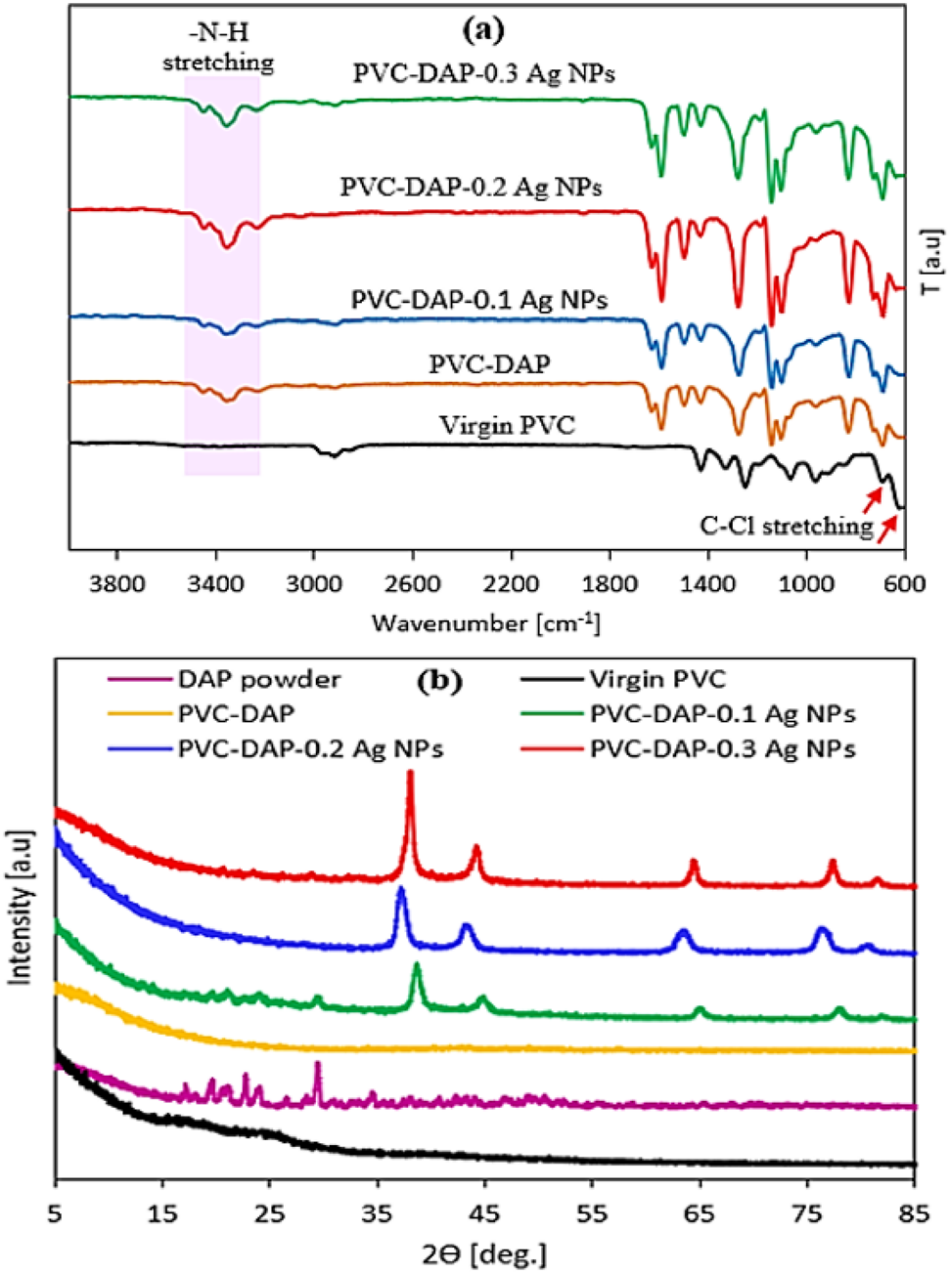
(a) FTIR spectra of virgin PVC, hybrid PVC, and the nanocomposites; (b) XRD patterns of pure DAP, virgin PVC, PVC-DAP, and nonabsorbent films containing varying quantities of Ag NPs.

Fig. 2(a) and (b) illustrate the zeta potential of synthesized Ag NPs and their particle size distribution metered using Malvern Zetasizer (Ver. 7.02).^6^ The stability of the Ag NPs in the solution was found to be good, as their zeta potential was a negative value (−19.6 mV), with a mean particle size distribution in the range of 30–35 nm, indicating uniform particle dispersion in the aqueous solution.^30^Fig. 3(a) demonstrates the FTIR spectra of virgin PVC and its hybrid by DAP in which the main absorption peaks for virgin PVC are located at ∼616 cm^−1^ and 648 cm^−1^ assigned to C–Cl stretching.^42^ Similarly, other absorption peaks were observed at ∼2912–2854 cm^−1^, corresponding to the symmetric and asymmetric vibrations of –CH and –CH_2_, respectively.^43,44^ For hybrid PVC, new characteristic peaks appeared at ∼3395–3225 cm^−1^, which signaled the NH- groups in DAP. Meanwhile, the disappearance of C–Cl peaks after the hybridization process was observed. All of them evidenced that the hybridization of PVC by DAP was achieved. For nanocomposite films, no significant changes were observed in the FTIR regions when Ag NPs were added, indicating the physical character of Ag NPs inside the polymer matrix. Additionally, ESI† about the hybridization process and chemical interaction between the PVA-DAP matrix and Ag NPs was provided by XRD patterns, as depicted in Fig. 3(b). Moreover, the spectrum attributed to virgin PVC and pure DAP was obtained for comparison with the PVC-DAP matrix. As depicted in Fig. 3(b), there were no sharp diffraction peaks in the XRD pattern for virgin PVC, evidencing its amorphous structure.^34,45^ However, sharp diffraction peaks with different intensities were observed for pure DAP at ∼2*θ* = 17.13°, 19.71°, 21.25°, 22.86°, 24.10°, and 29.60°, indicating its crystalline state.^23^ However, these peaks almost disappeared when DAP reacted with the PVC matrix to form the hybrid PVC-DAP, confirming that the hybridization of PVC by DAP was achieved. When incorporating Ag NPs into PVC-DAP, different peaks of 2*θ* appeared at ∼38.25°, 44.30°, 64.46°, 77.40°, and 81.02°, which were assigned to the Ag NPs in the matrix.^46,47^ Based on the Scherrer equation, it can also be observed that the crystallite size (*D*) of the indexed *hkl* (111) plane was estimated for PVC-DAP-0.1 Ag NPs, PVC-DAP-0.2 Ag NPs, and PVC-DAP-0.3 Ag NPs and was found to be 9, 11, and 17 Å, respectively. Thus, the fractional reduction in crystalline character and increase in crystallite size of Ag NPs in the XRD patterns of the polymeric membranes may indicate better incorporation and dispersion of Ag NPs into the membrane matrix. This result agrees well with the mechanical data.

### 3.2. Mechanical properties

The impacts of the Ag NP loadings on the tensile properties of hybrid PVC-DAP nanomembranes at different proportions (*i.e.*, 0.1, 0.2, and 0.3 wt%) are illustrated in Table 1. The mechanical parameters in terms of tensile strength at max (T.S., MPa), elongation at break (E.B., %), and Young's modulus (MPa) were measured under normal conditions. Virgin PVC was found to be a fairly flexible plastic with a tensile strength of 27.90 MPa and an elongation at break of ∼312%. By grafting PVC using dapsone, remark changes in both tensile strength and elongation at break were observed to be ∼20.45 MPa and 165%, respectively (Table 1). However, by adding 0.1 Ag NPs to the hybrid polymer, the ultimate strength started to increase to 21.80 MPa; then, a continuous increase was observed in the strength value by increasing the nanoparticle ratio (*i.e.*, 0.3%) to ∼25.40 MPs. Perhaps the reason behind the achievement of this property was attributed to the reinforcing effect and the good dispersion of Ag NPs inside the hybrid matrix, leading to better interfacial bonding between the components. These findings were evidenced by the SEM and FTIR data. Similarly, Young's modulus also increased as the quantity of Ag NPs increased. Nevertheless, the percentage of E.B. gradually reduced to ∼92.50% compared to unfilled polymer matrices (Table 1).

**Table 1:** Mechanical parameters derived from tensile testing for hybrid PVC-DAP absorbent films

Composite name	Virgin PVC	PVC-DAP	PVC-DAP-0.1% Ag NPs	PVC-DAP-0.2% Ag NPs	PVC-DAP-0.3% Ag NPs
T.S. at max (MPa)	27.90 ± 1.20	20.45 ± 1.62	21.80 ± 0.98	23.43 ± 1.84	25.36 ± 1.70
E.B. (%)	312.80 ± 3.85	165.40 ± 6.33	107.25 ± 4.82	102.90 ± 5.10	92.47 ± 3.18
Young's modulus (MPa)	177.50 ± 5.30	139.90 ± 4.70	211.95 ± 4.95	260.16 ± 6.23	277.30 ± 5.45

### 3.3. Adsorption analysis

#### 3.3.1. Effect of fabricated nanomembranes on the adsorption nature

**Fig. 4:**
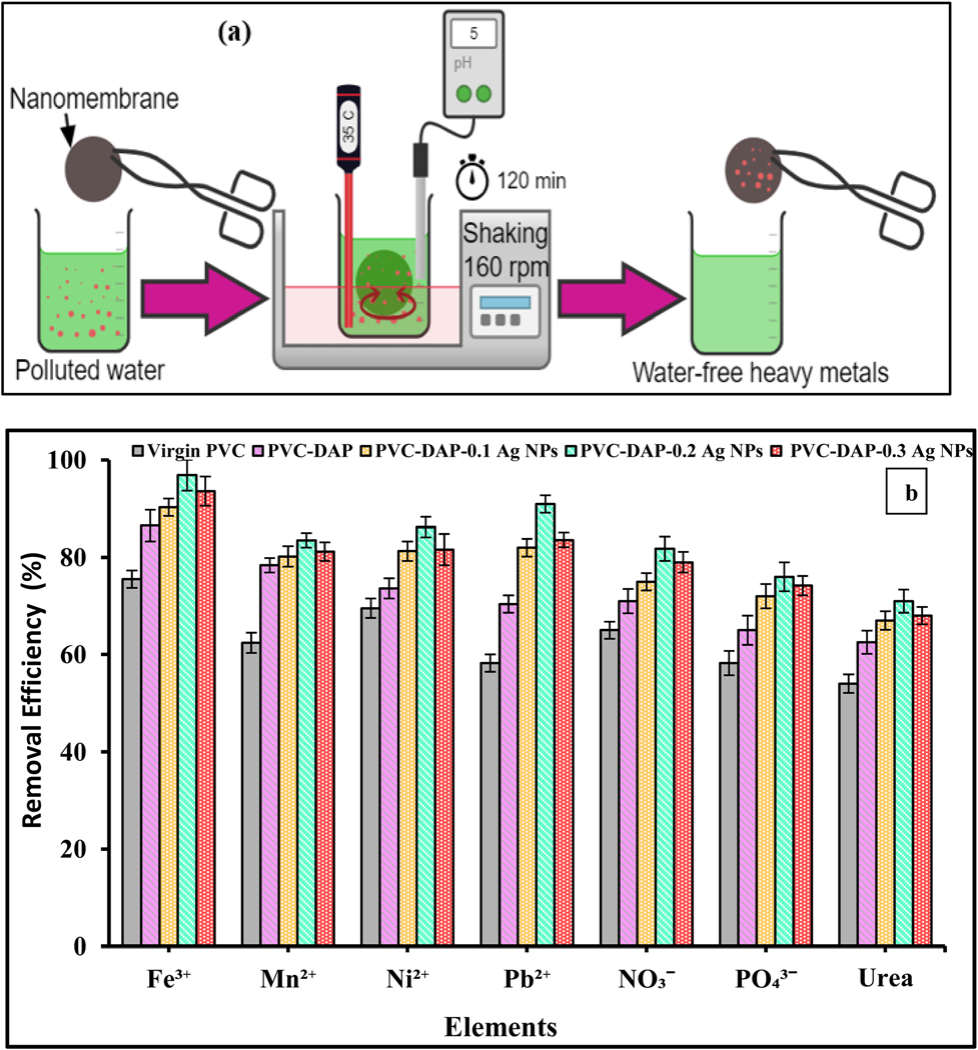
(a) Schematic representation of the batch adsorption test. (b) Effects of different membranes (virgin PVC, PVC-DAP, PVC-DAP-0.1 Ag NPs, PVC-DAP-0.2 Ag NPs, and PVC-DAP-0.3 Ag NPs) on the removal efficiency of the selected element ions (Fe^3+^, Mn^2+^, Ni^2+^, Pb^2+^, NO_3_^−^, PO_4_^3−^, and urea), element ion concentration 10 mg L^−1^, pH = 5–7.2 depending the metal ion type, time = 120 min, and adsorbent dose is 3 g L^−1^, at 35 °C.

The adsorbent dosage is one of the key parametric variables that affects the adsorption process. The primary goal of this study is to develop hybrid polyvinyl chloride-dapsone-based nanomembranes by embedding three different concentrations (0.1, 0.2, and 0.3) of Ag NPs to improve membrane performance for water purification. The effects of five different membranes (virgin PVC, PVC-DAP, PVC-DAP-0.1 Ag NPs, PVC-DAP-0.2 Ag NPs, and PVC-DAP-0.3 Ag NPs) on the removal of the selected elements (Fe^3+^, Mn^2+^, Ni^2+^, Pb^2+^, NO_3_^−^, PO_4_^3−^, and urea) are shown in Fig. 4(b). All created improved membranes (virgin PVC, PVC-DAP, PVC-DAP-0.1 Ag NPs, PVC-DAP-0.2 Ag NPs, and PVC-DAP-0.3 Ag NPs) were assessed in terms of element removal at 35 °C (Fig. 4(b)). When compared to the virgin PVC membrane, it can be observed that all modified PVC-DAP membranes showed significantly higher removal efficiency. It was evident that the PVC-DAP-0.2 Ag NP membrane was viewed as the optimal dose at 120 min of contact time. Using 10 g L^−1^ element ion concentrations, the PVC-DAP-0.2 Ag NP membrane demonstrated removal efficiencies of 95, 83.5, 86.2, 90.9, 81.8, 76, and 71 for Fe^3+^, Mn^2+^, Ni^2+^, Pb^2+^, NO_3_^−^, PO_4_^3−^, and urea, respectively. A dose of 0.2 wt% of Ag NPs in the matrix resulted in the highest level of adsorption effectiveness. The removal effectiveness (%) of the chosen element ions was demonstrated to steadily increase as the concentration of Ag NPs increased from PVC-DAP-0.1 to PVC-DAP-0.2. This was caused by an increase in the number of functional groups that were inserted, such as sulfonyl (O=S=O), amine (–NH), and Ag NPs, which enhanced the number of active spots for element ion binding. The effectiveness of the removal was reduced as the Ag NP content increased further to 0.3 wt%. The interfacial debonding between the Ag NPs and the PVC-DAP matrix caused by nanofiller aggregation was likely the cause, which reduced the membrane's reactivity. This result was consistent with that previously established for the adsorption of various ions using magnetic graphene oxide (mGO) NPs.^6^ Regarding the adsorption mechanism, many variables, such as pH, ionic strength, and the surface nature of the adsorbent (functional groups' active sites and surface area), can influence the adsorption mechanism between the PVC-DAP-0.2 Ag NP membrane and the selective elements (Fig. 1(a) and 4(a)). The adsorption of Fe^3+^, Mn^2+^, Ni^2+^, Pb^2+^, NO_3_^−^, PO_4_^3−^, and urea ions was controlled by surface complexations, ion exchange, and electrostatic attraction using a PVC-DAP-0.2 Ag NP membrane. Reactive functional groups in the PVC-DAP-0.2 Ag NP membrane absorbed negatively chartered phosphate and nitrate ions through the electrostatic force of attraction.^48^ During adsorption, the amine (–NH_2_) and oxygenated group ions found in the PVC-DAP-0.2 Ag NP membrane replace the harmful ions in the water through the mechanisms of ion exchange and electrostatic attraction.^49^ Additionally, aqueous pollutants may be pulled to the PVC-DAP-0.2 Ag NP membrane through chelating, complexation, van der Waals forces, electrostatic interactions, and/or hydrogen bonding.^50^ The hydrophilic surfaces of PVC-DAP-0.2 Ag NP membrane have amine and oxygenated groups that can easily be deprotonated to a negative charge to attract positively charged heavy metals, depending on the pH of the solution.

#### 3.3.2. SEM analysis

**Fig. 5:**
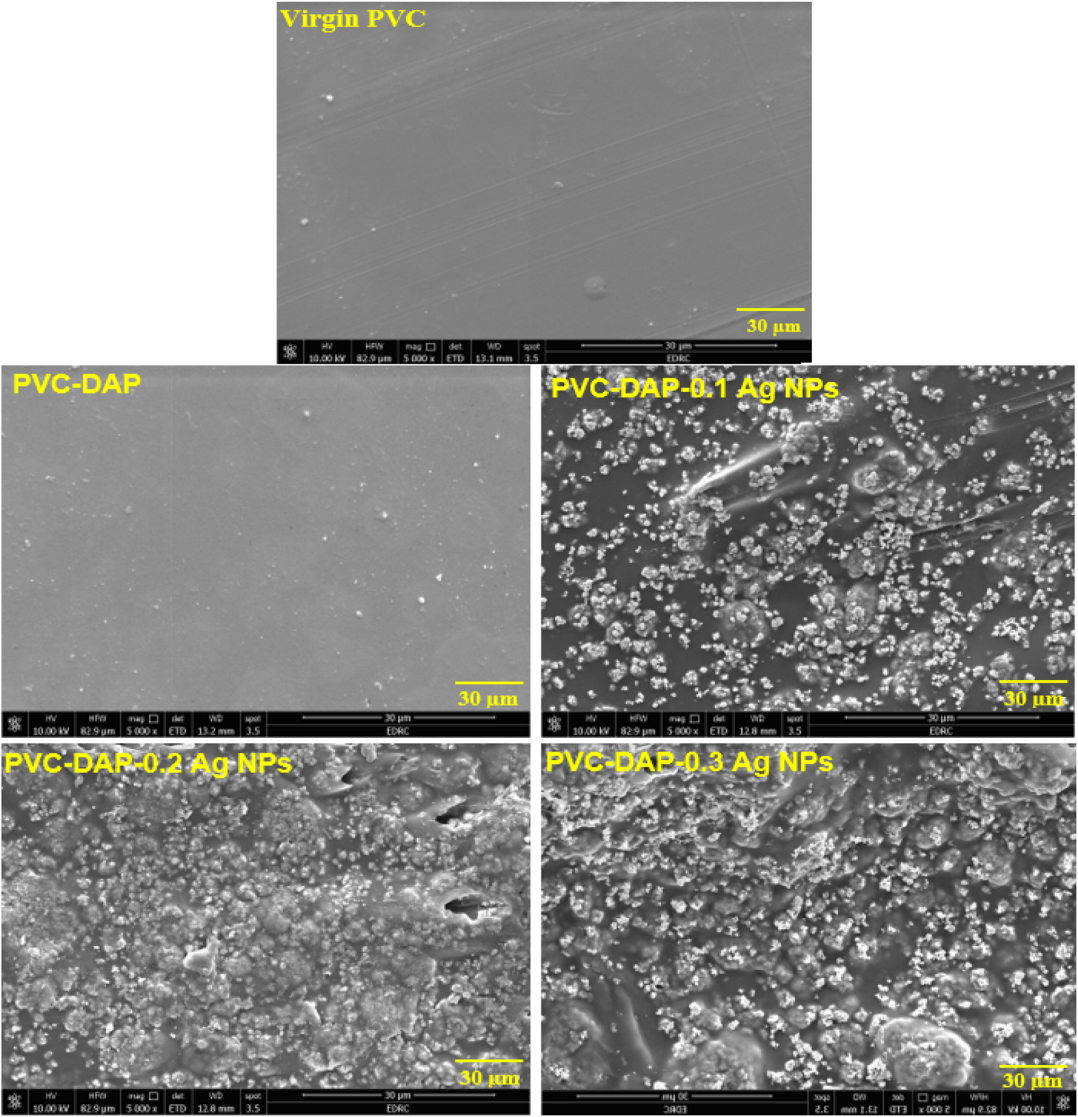
SEM images of virgin PVC, PVC-DAP and the nanocomposites containing various Ag NP proportions after adsorption at 5000×.

SEM images exhibited the surface morphology of the fabricated membranes after adsorption and the impact of Ag NPs on the metal ion removal efficiency inside the hybrid PVC matrix, as depicted in Fig. 5. It was obviously observed that the pores and surfaces of adsorbent-based Ag NPs were covered by metal ions, resulting in rough and porous surfaces. Moreover, it was found that the adsorption capacity of the PVC-DAP-0.2 Ag NP membrane increased compared to that of the unfilled matrices or other filled nanomembranes. This outcome was supported by SEM-EDX observations, as shown in Fig. 10.

#### 3.3.3. BET evaluation

**Fig. 6:**
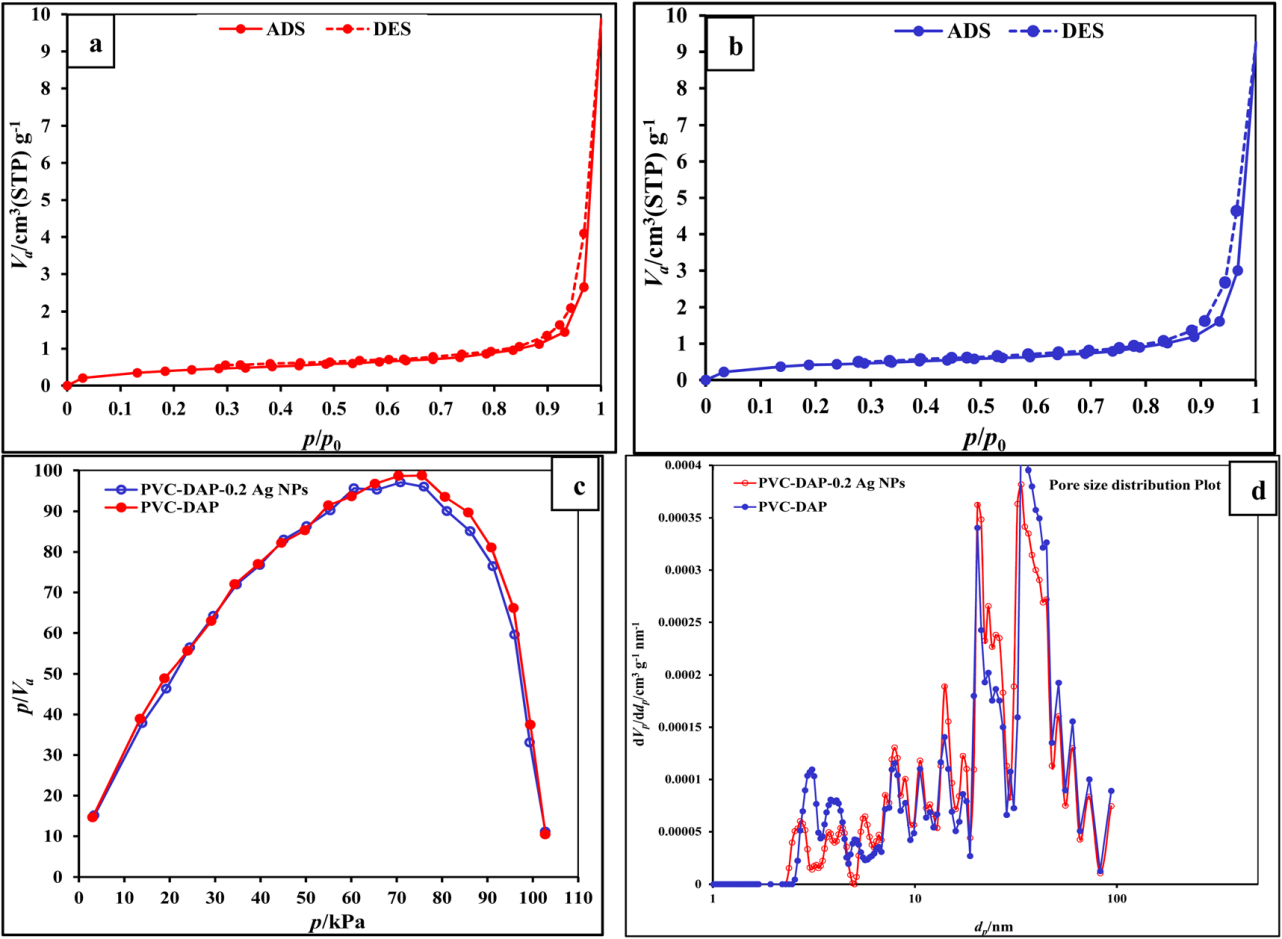
(a and b) N_2_ adsorption–desorption isotherm; (c) pore size distribution; (d) BET pore area and pore volume for PVC-DAP and PVC-DAP-0.2 Ag NP membranes.

The BET method is widely used to determine the porosity and surface area of constituents that are mesoporous and microporous. The two most important parameters in membrane fields are surface area and porosity, which indicate the membrane's functional characteristics and may even indicate potential locations for foulant build-up. The PVC-DAP and PVC-DAP-0.2 Ag NP membranes, as shown in Fig. 6a and b, had type IV isotherms with type H_3_ hysteresis loops that were in line with the IUPAC classification. Through capillary condensation, hysteresis loops were connected to a wide variety of physisorption isotherms. The presence of a hysteresis loop associated with the filling and clearing of the adsorbent characterizes the Type IV isotherm, which marks the beginning of multilayer formation. When the porous constituents have mesopores, the IV isotherm is common (Fig. 6a and b). The membrane porosity increased as a result of the addition of Ag NPs to the PVC-DAP matrix. Three categories of pore sizes can be distinguished based on pore width: macropore (>50 nm), mesopore (2–50 nm), and micropore (<2 nm). As shown in Table 2 and Fig. 6c and d, it was found that the addition of Ag NPs to the PVC-DAP material backbone gradually enhanced the BET surface area, increasing the pore volume and pore size. N_2_ adsorption/desorption isotherms were used to test the PVC-DAP and PVC-DAP-0.2 Ag NP membrane's specific surface area and mean pore diameter. The PVC-DAP membrane's specific surface area and mean median pore diameter were measured to be approximately 20 m^2^ g^−1^ and 52 nm, respectively. In contrast, the PVC-DAP-0.2 Ag NP membrane had these values measured to be 25 m^2^ g^−1^ and 65 nm, respectively. These results showed that the investigated membrane performance improved.

**Table 2:** BET measurements of PVC-DAP and PVC-DAP-0.2 Ag NPs

Element	PVC-DAP	PVC-DAP-0.2 Ag NPs
BET surface area [m^2^ g^−1^]	1.5395	1.5569
Total pore volume [cm^3^ g^−1^]	0.011316	0.011656
*a*_s_, Lang [m^2^ g^−1^]	20.00	25.00
Average pore diameter [nm]	20.36	30.29
Median pore diameter [nm]	52.00	65.00

#### 3.3.4. Effect of initial concentration of selected ions and analysis of adsorption isotherms

**Fig. 7:**
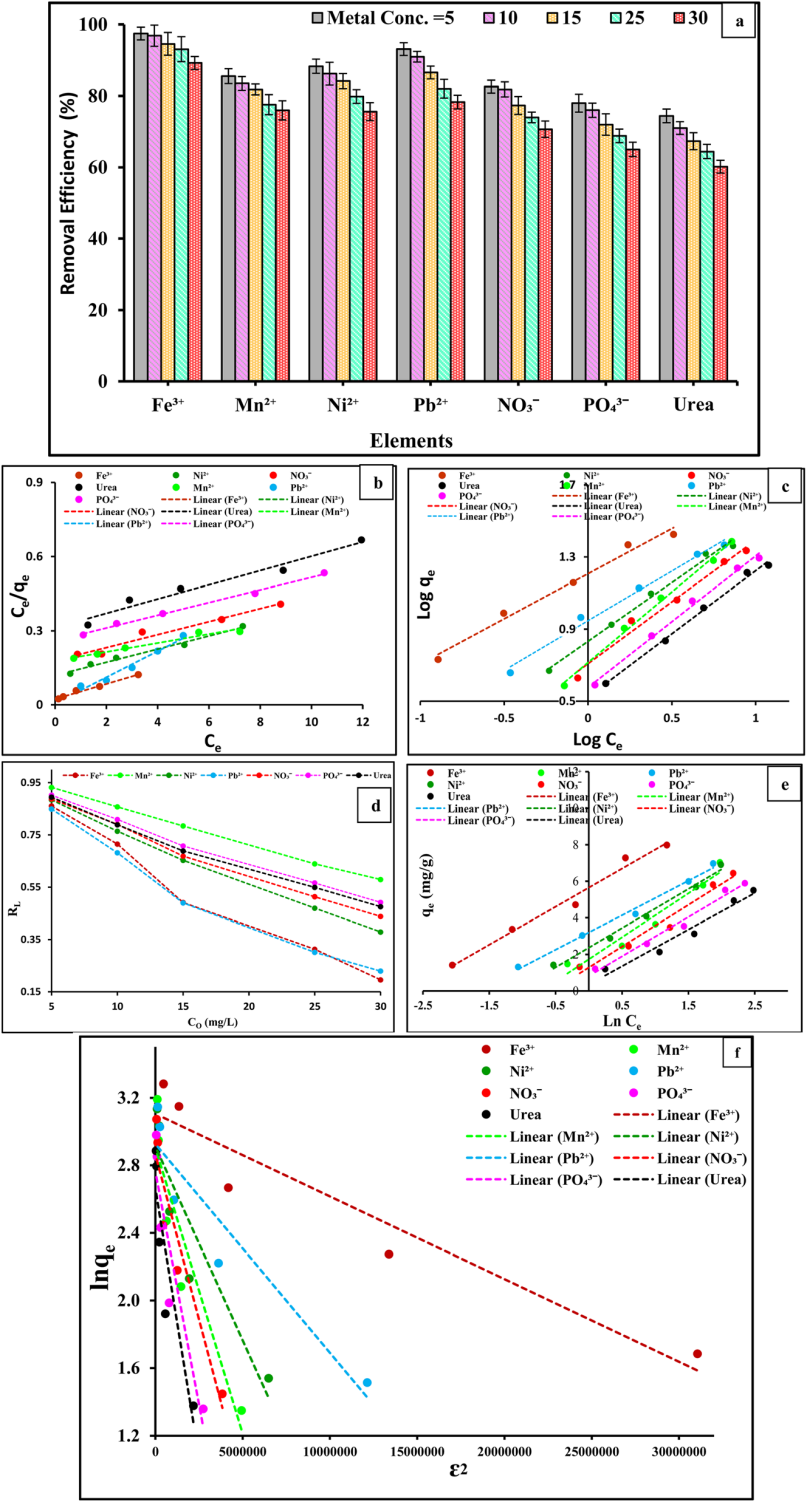
(a) Effects of element ion concentration (ranging from 5 to 30 mg L^−1^); (b) linearized Langmuir; (c) Freundlich; (d) effect of concentration on Langmuir separation factor; (e) Temkin models; and (f) D–R adsorption isotherm models for Fe^3+^, Mn^2+^, Ni^2+^, Pb^2+^, NO_3_^−^, PO_4_^3−^, and urea ions onto the PVC-DAP-0.2 Ag NP membrane.

The effects of concentrations on Fe^3+^, Mn^2+^, Ni^2+^, Pb^2+^, NO_3_^−^, PO_4_^3−^, and urea adsorption capability onto the PVC-DAP-0.2 Ag NP membrane were obtained, as illustrated in Fig. 7(a). The amount and impact of the mass resistance transfer of metallic ions between the aqueous and solid phases were overcome by the energy force offered by the concentration of metal ions.^51^ The percentage removal of the studied ions decreased as the concentrations of Fe^3+^, Mn^2+^, Ni^2+^, Pb^2+^, NO_3_^−^, PO_4_^3−^, and urea increased from 5 to 30 mg L^−1^, as observed in the plot. This finding suggests that the insufficient number of active coverage sites of the adsorbent became saturated at higher concentrations. Consequently, the concentration of metal ions at equilibrium increased as the capacity for heavy metal uptake increased. These findings suggest that the real amount of Fe^3+^, Mn^2+^, Ni^2+^, Pb^2+^, NO_3_^−^, PO_4_^3−^, and urea ions adsorbed increased with the metal ion concentration per unit mass of the adsorbent. The adsorption capacity was calculated for the selective elements (Fe^3+^, Mn^2+^, Ni^2+^, Pb^2+^, NO_3_^−^, PO_4_^3−^, and urea), as shown in Table 3. By increasing the initial element ion concentrations, the ratio of moles of ions to the freely available surface area of the PVC-DAP-0.2 Ag NP membrane became higher, which was the cause of the reduced removal%. Similar outcomes were described for heavy metal adsorption onto Zeo/PVA/SA NC beads.^39^

**Table 3:** Adsorption capacity of the Fe^3+^, Mn^2+^, Ni^2+^, Pb^2+^, NO_3_^−^, PO_4_^3−^, and urea element ions using PVC-DAP-0.2 Ag NP membrane

Element concentrations	Adsorption capacity (*q*_e_; mg g^−1^)						
	Fe^3+^	Mn^2+^	Ni^2+^	Pb^2+^	NO_3_^−^	PO_4_^3−^	Urea
5	0.160	0.048	0.043	0.241	0.196	0.115	0.290
10	0.423	0.167	0.105	0.550	0.460	0.302	0.607
15	0.744	0.362	0.273	0.910	0.793	0.673	1.133
25	1.441	1.011	0.579	1.867	1.680	1.499	2.167
30	2.014	1.485	1.081	2.403	2.440	2.170	2.933

The isotherms for adsorption describe the relationship between adsorbate concentration and their deposition onto the adsorbent surface and calculate the adsorbent's capacity for adsorption, making them crucial for adsorption investigations. Comparing various sorbents under various conditions for experimentation requires the analysis of equilibrium data on a particular adsorption isotherm. Therefore, it was crucial to determine the best correlation for the equilibrium curves. The equilibrium properties of the adsorption of metal ions in aqueous solutions were modelled using various two-parameter equations, such as those of Langmuir,^52^ Freundlich,^52^ Temkin,^53^ and Redlich–Peterson,^54^ and Table S1† presents the equations. Fig. 7(b)–(f), respectively, illustrates the Langmuir, Freundlich, Temkin, and Redlich–Peterson adsorption isotherms for Fe^3+^, Mn^2+^, Ni^2+^, Pb^2+^, NO_3_^−^, PO_4_^3−^, and urea at pH 5–7.2 according to the type of metal ions. Table 4 illustrates a list of the different adsorption isotherm model parameters and the linear regression coefficients, *R*^2^. It can be observed that the *R*^2^ values were much closer to unity for the Freundlich and Temkin models than for the other two. Therefore, the Temkin and Freundlich models can best describe the adsorption data of Fe^3+^, Mn^2+^, Ni^2+^, Pb^2+^, NO_3_^−^, PO_4_^3−^, and urea ions on the PVC-DAP-0.2 Ag NP membrane in the examined concentration range. The adsorption of Fe^3+^, Mn^2+^, Ni^2+^, Pb^2+^, NO_3_^−^, PO_4_^3−^, and urea onto PVC-DAP-0.2 Ag NP membrane was best described by the Freundlich isotherm, suggesting that multilayer adsorption rather than adsorption onto a uniform site occurred in the system of the PVC-DAP-0.2 Ag NP membrane-metal ions. It was concluded that the surface of the adsorbent changed from a homogeneous surface to a heterogeneous surface as the concentration of metal ions in the sample increased. This results in the adsorbent having a multilayer adsorption effect and interacting strongly with the ions Fe^3+^, Mn^2+^, Ni^2+^, Pb^2+^, NO_3_^−^, PO_4_^3−^, and urea on its surface. However, the PVC-DAP-0.2 Ag NP membrane's surface pores and cracks were filled with these ions, increasing the efficiency of combining active sites on the surface of the adsorbent with Fe^3+^, Mn^2+^, Ni^2+^, Pb^2+^, NO_3_^−^, PO_4_^3−^, and urea ions. This resulted in an improvement in the adsorption capacity of the adsorbent for these ions. The ion concentration on the surface of the adsorbent increased, the critical energy of adsorption increased, and the attraction between metal ions enhanced, all of which can further increase layer adsorption capacity, as shown in the analysis in Fig. 7(c), which demonstrated that the heterogeneity constant of the Freundlich model was greater than 1. Although the *R*^2^ for Langmuir was higher than 0.96 for all metal ions, monolayer adsorption also played a significant role in the adsorption of the metal ions onto the PVC-DAP-0.2 Ag NP membrane. The PVC-DAP-0.2 Ag NP membrane was a favorable absorbent to remove Fe^3+^, Mn^2+^, Ni^2+^, Pb^2+^, NO_3_^−^, PO_4_^3−^, and urea ions from aqueous solution because the values of the equilibrium parameter, RL, were in the range of 0 < RL < 1. The adsorption approach was favorable because the calculated RL value was less than 1 and the value of n was more than 1. The adsorption energy (*E*) obtained from the Dubinin–Radushkevich (D–R) isotherm was higher at 8 kJ mol^−1^, suggesting that the uptake of Al^3+^, Fe^3+^, Cr^2+^, Mn^2+^, Ni^2+^, Pb^2+^, NO_3_^−^, PO_4_^3−^, and urea onto PVC-DAP-0.2 Ag NP membranes was by chemisorption. The increased bonding energy (*E*, kJ mol^−1^) values found in this investigation support a chemical interaction mechanism for the interaction of the PVC-DAP-0.2 Ag NP membrane with metal ions. The isotherm constants (*q*_m_ and *β*) for the adsorption of metal ions were determined from the linear form of the D–R model, and the results are shown in Table 4. The constant values in Table 4 show that for each metal ion, the maximum sorption capacity increased as the temperature increased. A chemical phenomenon was also involved in the adsorption of Fe^3+^, Mn^2+^, Ni^2+^, Pb^2+^, NO_3_^−^, PO_4_^3−^, and urea according to the mean free energies. In general, Redlich–Peterson constant (*β*) values typically ranging from 0 to 1 indicate a favorable adsorption.^55^ The plots for this isotherm are showcased in Fig. 7(f). Values of *β* ((mol^2^ K^−2^ J^−2^) × 100 000) ranged from 0.82 to 0.91 (Table 4), therefore indicating that all of the ions were favorably adsorbed by the PVC-DAP-0.2 Ag NP membrane. Furthermore, the *R*^2^ values were closer to unity for the Redlich–Peterson and Freundlich models than for the other models. Therefore, the equilibrium adsorption data for Fe^3+^, Mn^2+^, Ni^2+^, Pb^2+^, NO_3_^−^, PO_4_^3−^, and urea ion adsorption on PVC-DAP-0.2 Ag NP membrane can be represented more appropriately by the Temkin and Freundlich models in the concentration range under study.

**Table 4:** Results obtained from Langmuir, Freundlich, Temkin, and D–R adsorption isotherm models for Fe^3+^, Mn^2+^, Ni^2+^, Pb^2+^, NO_3_^−^, PO_4_^3−^, and urea element ions onto the PVC-DAP-0.2 Ag NP membrane

Parameters	Fe^3+^	Mn^2+^	Ni^2+^	Pb^2+^	NO_3_^−^	PO_4_^3−^	Urea
**Langmuir**							
*q*_m,calc_ (mg g^−1^)	32.9	55.6	37.5	18.9	38.2	39.1	34.6
*K*_L_	1.27	0.10	0.22	0.52	0.15	0.10	0.09
*R* ^2^	0.986	0.963	0.985	0.974	0.962	0.995	0.963
*q*_e,exp_	26.60	24.30	23.00	23.20	21.60	19.7	17.90

**Freundlich**							
*n*	2.00	1.28	1.54	1.81	1.46	1.37	1.43
*K*_f_ (mg g^−1^)	16.11	5.18	6.80	8.84	5.10	3.77	3.33
*R* ^2^	0.985	0.996	0.992	0.988	0.982	0.994	0.995

**Temkin models**							
*B*_1_ (J mol^−1^)	2.082	2.406	2.146	1.886	2.280	2.153	2.00
*A* (L mol^−1^)	15.110	2.054	3.012	5.461	1.759	1.461	1.192
*R* ^2^	0.982	0.950	0.990	0.991	0.967	0.983	0.963

**Dubinin–Radushkevich**							
*q*_m_ (mg g^−1^)	22.30	18.26	18.39	18.67	17.54	15.79	14.28
*E* (KJ mol^−1^)	3162	1291	1581	2236	1118	1000	913
*β* (mol^2^ K^−2^ J^−2^) × 100 000	0.914	0.869	0.853	0.893	0.895	0.868	0.825
*R* ^2^	0.914	0.869	0.853	0.893	0.895	0.868	0.825

#### 3.3.5. Effect of temperature on the selected element ions and analysis of thermodynamics parameters

**Fig. 8:**
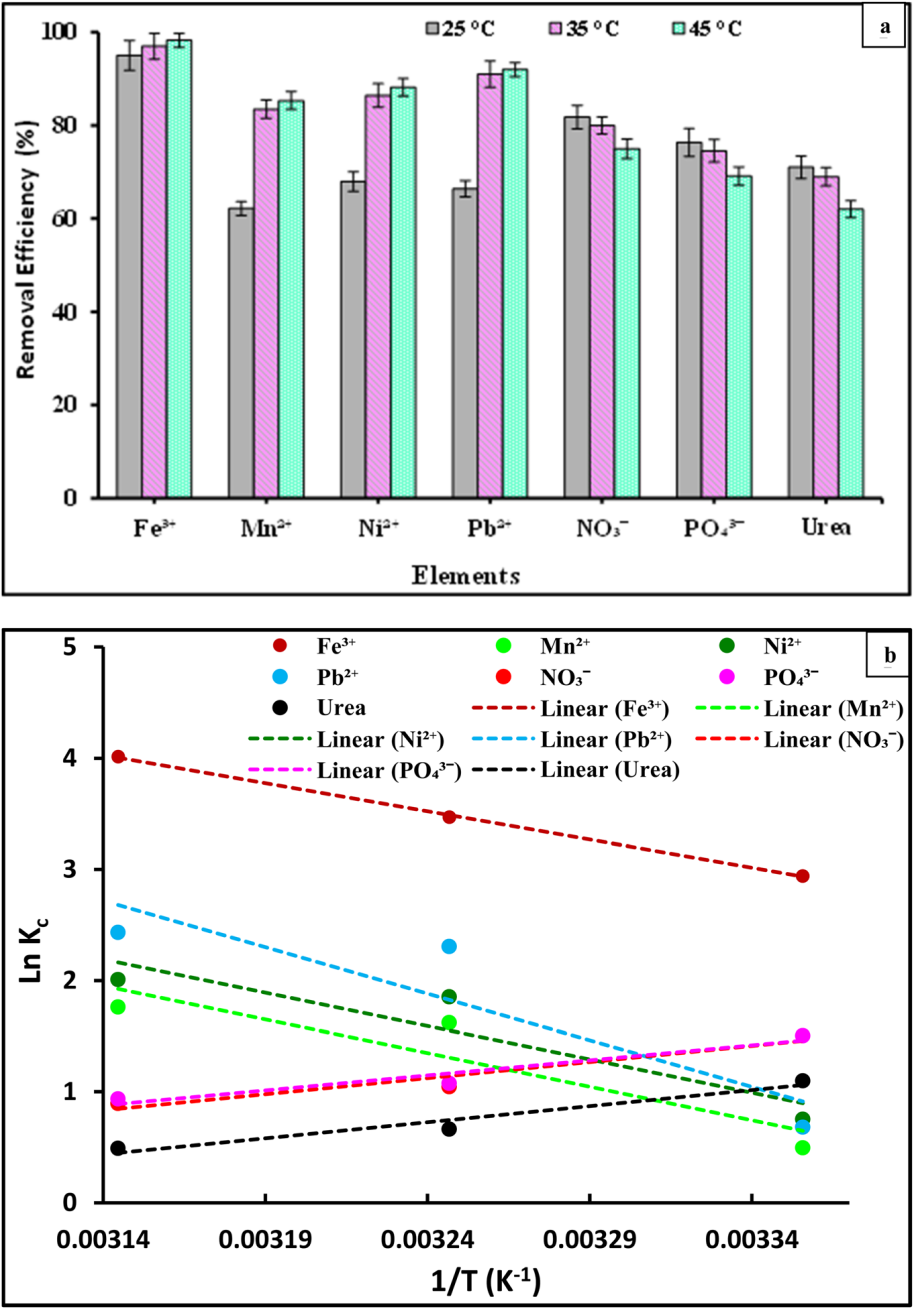
(a) Effect of temperature; (b) Van't Hoff plot of ln *K*_c_*versus* 1/*T* for on adsorption of Fe^3+^, Mn^2+^, Ni^2+^, Pb^2+^, NO_3_^−^, PO_4_^3−^, and urea element ions onto the PVC-DAP-0.2 Ag NP membrane, ion concentration 10 mg L^−1^, pH = 5–7.2 according to the type of metal ions, time = 120 min., and adsorbent dose is 3 g L^−1^.

The effect of temperature on Fe^3+^, Mn^2+^, Ni^2+^, Pb^2+^, NO_3_^−^, PO_4_^3−^, and urea element ion adsorption by PVC-DAP-0.2 Ag NP membrane was examined using 10 mg L^−1^ ion concentrations. At three temperatures 25, 35, and 45 °C, the effect of temperature on the adsorption was tested, as shown in Fig. 8(a). The temperature range used in this experiment was based on the recorded thermal groundwater temperature that was measured at the field site (18–36.8 °C) in May 2022 according to Eissa 2023.^56^ The results of the experiment showed that as the temperature increased from 25 °C to 45 °C, the removal efficiency improved in the case of Fe^3+^, Mn^2+^, Ni^2+^, and Pb^2+^ ions. This observation was consistent with that conducted elsewhere.^57,58^ The mobility and penetration of metal ions within the adsorbent's porous structure might be facilitated by the higher temperature, which was the reason for this phenomenon. The kinetic energy of the metal ions increases as the temperature increases, improving their ability to move through the adsorbent material's pores. The energy barrier, often referred to as the activation energy that metal ions must cross to be adsorbed, is removed by this enhanced mobility.^57^

This suggests that the sorption process of the Fe^3+^, Mn^2+^, Ni^2+^, and Pb^2+^ ions is endothermic in character. Furthermore, as the temperature increased, the number of active sites on the PVC-DAP-0.2 Ag NP membrane increased for adsorption. This observation was the result of a change in the pore size within the PVC-DAP-0.2 Ag NP membrane's internal structure, which made it easier for the removal of metal ions. This demonstrated that the temperature significantly affected the adsorption process in terms of the rate at which adsorbate chemicals diffuse and the size of the pores within the adsorbent materials. Consequently, increasing the temperature caused the diffusion rate to increase, resulting in a decrease in the solution's viscosity.^59,60^

The efficiency of removing metal ions from the PVC-DAP-0.2 Ag NP membrane for investigation increases as the temperature increases. It is obvious from Fig. 8(a) that the adsorption of NO_3_^−^, PO_4_^3−^, and urea ions was negatively impacted by the increase in temperature. This suggested that the sorption process of the NO_3_^−^, PO_4_^3−^, and urea ions was exothermic in character, and this result was supported by previously conducted studies.^61–63^ The experimental data and relevant equations were used to analyzed the thermodynamic parameters Δ*G*° (Gibbs free energy, J mol^−1^), Δ*H*° (enthalpy of the system, J mol^−1^), and Δ*S*° (entropy, J mol^−1^ K^−1^) of the adsorption process of the Fe^3+^, Mn^2+^, Ni^2+^, Pb^2+^, NO_3_^−^, PO_4_^3−^, and urea element ions (Table S1†). The slope and intercept of the plot of ln *K*_c_*vs.* 1/*T* (Fig. 8(b)), which demonstrated linearity with good correlation coefficient values (*R*^2^), were used to determine Δ*H*° and Δ*S*°. The values of the thermodynamic parameters Δ*G*°, Δ*H*°, and Δ*S*° at various temperatures are shown in Table 5. The negative values of Δ*G*° showed that the Fe^3+^, Mn^2+^, Ni^2+^, Pb^2+^, NO_3_^−^, PO_4_^3−^, and urea element ion adsorption onto the PVC-DAP-0.2 Ag NP membrane was feasible and spontaneous in nature. The Fe^3+^, Mn^2+^, Ni^2+^, and Pb^2+^ ions appear to be more easily absorbed as temperature increases, whereas NO_3_^−^, PO_4_^3−^, and urea ions appear to be less absorbable as temperature increases according to the values of Δ*G*° (Table 5). The value of Δ*G*°, which increases (NO_3_^−^, PO_4_^3−^, and urea) and decreases (Fe^3+^, Mn^2+^, Ni^2+^, and Pb^2+^) with temperature showed that the adsorption procedure was favorable at low and high temperatures, respectively. The NO_3_^−^, PO_4_^3−^, and urea ions were adsorbed onto the PVC-DAP-0.2 Ag NP membrane with negative values for Δ*H*° and Δ*S*°, demonstrating the exothermic nature of the adsorption process controlled by physical adsorption. Randomness decreased at the solid/liquid interface throughout the adsorption procedure, reflecting the DAP-0.2 Ag NP membrane's high affinity for the NO_3_^−^, PO_4_^3−^, and urea ions. The endothermic nature of the adsorption process and the increase in randomness at the solid–liquid interface during the adsorption operation were suggested by the negative values of Δ*H*° and Δ*S*° for the Fe^3+^, Mn^2+^, Ni^2+^, and Pb^2+^ ions. Δ*H*° values obtained for the adsorption of Fe^3+^, Mn^2+^, Ni^2+^, and Pb^2+^ ions onto the DAP-0.2 Ag NP membrane were also shown to be higher than 40 kJ mol^−1^, indicating chemical adsorption.

**Table 5:** Thermodynamic parameters for Fe^3+^, Mn^2+^, Ni^2+^, Pb^2+^, NO_3_^−^, PO_4_^3−^, and urea element ions onto the PVC-DAP-0.2 Ag NP membrane

Element ions	Thermodynamic parameters					
	Δ*H* (J mol^−1^)	Δ*S* (J mol^−1^ K^−1^)	Δ*G* (J mol^−1^)			*R* ^2^
			298	308	318	
Fe^3+^	32 725	136	−7766	−9125	−10484	0.963
Mn^2+^	50 216	174	−1605	−3344	−5083	0.845
Ni^2+^	49 886	175	−2222	−3970	−5719	0.855
Pb^2+^	69 689	241	−2260	−4674	−7089	0.819
NO_3_^−^	−24052	−69	−3609	−2923	−2237	0.932
PO_4_^3−^	−22516	−63	−3618	−2984	−2350	0.930
Urea	−24096	−72	−2626	−1905	−1185	0.951

#### 3.3.6. Effect of contacting time on the selected element ions and analysis of adsorption kinetics

**Fig. 9:**
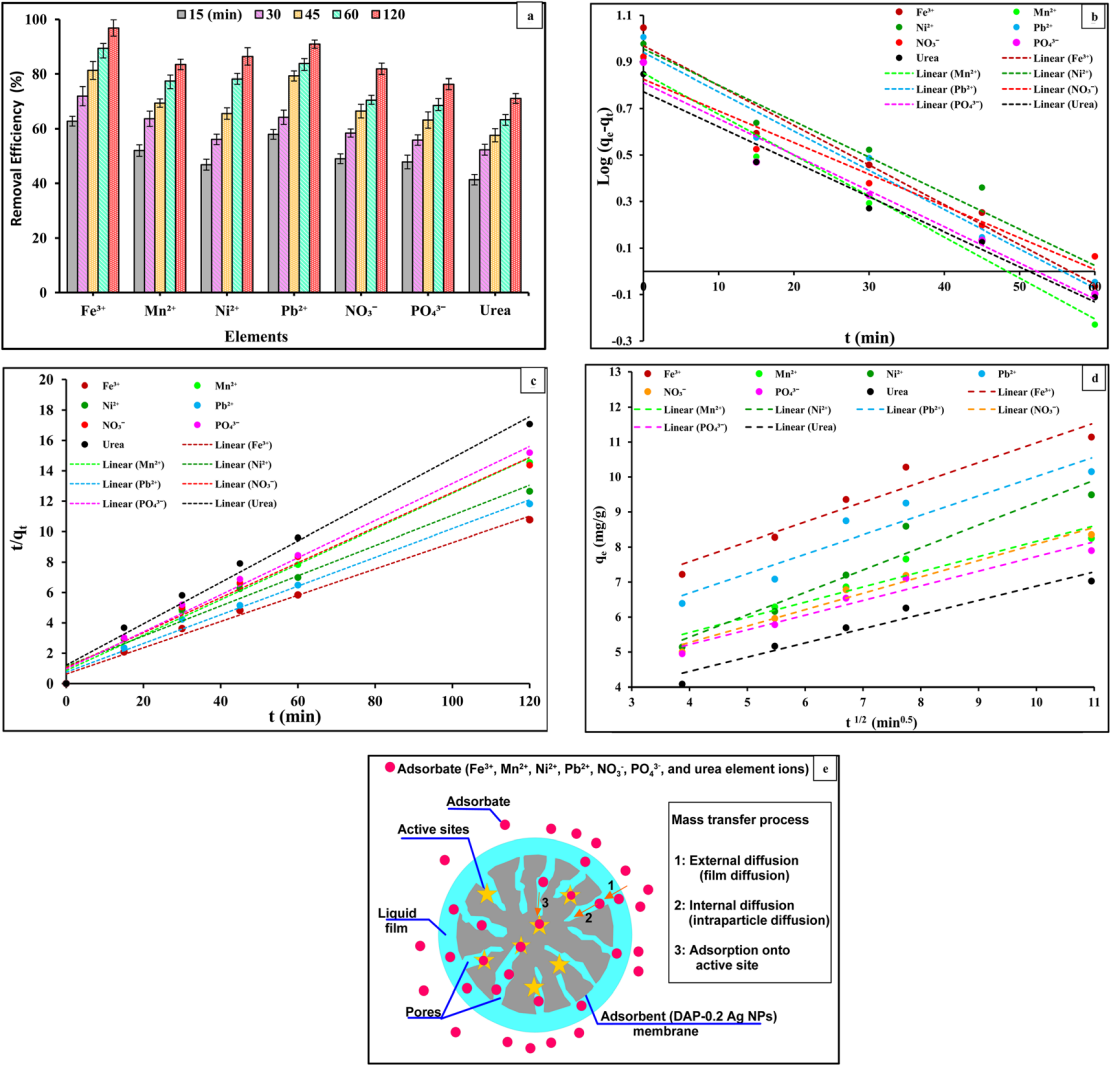
(a) Effect of contacting time; (b) pseudo first order kinetic; (c) pseudo second order; (d) intraparticle diffusion adsorption kinetic models obtained from the experimental data of Fe^3+^, Mn^2+^, Ni^2+^, Pb^2+^, NO_3_^−^, PO_4_^3−^, and urea ions onto PVC-DAP-0.2 Ag NP membrane, metal ions concentration 10 mg L^−1^, pH = 5–7.2, time ranging from 0 to 120 min, adsorbent dose is 3 g L^−1^ and temp. = 35 °C; and (e) adsorption kinetics mass transfer procedures.

The adsorption levels of Fe^3+^, Mn^2+^, Ni^2+^, Pb^2+^, NO_3_^−^, PO_4_^3−^, and urea element ions onto the PVC-DAP-0.2 Ag NP membrane as a function of interaction time are presented in Fig. 9(a). The results of the experiment's plot showed that the adsorption rate of the element ions was initially high and subsequently decreased as equilibrium approached. Consequently, the sorption capacity of the DAP-0.2 Ag NP membrane increases with time. This suggests that as the adsorbate species diffuse from the bulk solution to the adsorbent surface, the number of pores on the adsorbent's active binding centers increases, reducing the mobility and availability of Fe^3+^, Mn^2+^, Ni^2+^, Pb^2+^, NO_3_^−^, PO_4_^3−^, and urea element ions into the DAP-0.2 Ag NP membrane media. Quick adsorption may be due to contacts of Fe^3+^, Mn^2+^, Ni^2+^, Pb^2+^, NO_3_^−^, PO_4_^3−^, and urea element ions with obtainable surface adsorption spots onto the DAP-0.2 Ag NP membrane, whereas the subsequent gradual adsorption may be caused by the selective element being taken up into the pores of the adsorbents. Equilibrium was reached at 45 min for Fe^3+^, Mn^2+^, Ni^2+^, Pb^2+^, PO_4_^3−^, and urea element ions and at 60 min for NO_3_^−^ ions.

One of the key factors determining an adsorbent's efficacy was its adsorption kinetics, which characterized the solute absorption rate by regulating the diffusion process and the residence duration of an adsorbate at the solid/solution interface. Based on the experimental results, the linear form pseudo-1st-order and pseudo-2nd-order kinetic models were studied to determine the kind of adsorption mechanism present in the experimental system. The linear forms of pseudo-1st-order and pseudo-2nd-order kinetics models of the adsorption of the Fe^3+^, Mn^2+^, Ni^2+^, Pb^2+^, NO_3_^−^, PO_4_^3−^, and urea element ions are presented in Fig. 9(b) and (c). The intercepts and slopes of Fig. 9(b) and (c) were used to compute the parameters of both kinetic models (Table 6). The pseudo-2nd-order model fits the experimental data more accurately than the pseudo-1st-order model. The high *R*^2^ values of the pseudo-2nd-order model suggested that the chemical adsorption process might occur between the DAP-0.2 Ag NP membrane and Fe^3+^, Mn^2+^, Ni^2+^, Pb^2+^, NO_3_^−^, PO_4_^3−^, and urea element ions. A better correlation (Fig. 9(b) and (c)) was confirmed by pseudo-2nd-order model theory for the experimental data, followed by intra-particle diffusion for the adsorption of Fe^3+^, Mn^2+^, Ni^2+^, Pb^2+^, NO_3_^−^, PO_4_^3−^, and urea element ions on the surface of the adsorbent species (DAP-0.2 Ag NP membrane) identified with high linear regression (*R*^2^) values. The removal of Fe^3+^, Mn^2+^, Ni^2+^, Pb^2+^, NO_3_^−^, PO_4_^3−^, and urea element ions *via* chemical adsorption, which was the rate-limiting mechanistic step, was thus suggested to involve a boundary layer effect on the DAP-0.2 Ag NP membrane substrate. This effect results from the interaction of physicochemical properties between the adsorbent and adsorbate phases. The estimated *q*_e_ values were nearly identical to the experimentally measured values, indicating the kinetic theory's suitability for the adsorption of the Fe^3+^, Mn^2+^, Ni^2+^, Pb^2+^, NO_3_^−^, PO_4_^3−^, and urea element ions onto the DAP-0.2 Ag NP membrane under study. Additionally, the computed equilibrium capacities *q*_e (cal.)_ from the pseudo-2nd-order model had values that agreed well with the experimental q values *q*_e (exp)_. The intraparticle diffusion (IPD) kinetic model was employed to further study the possibility that distinct adsorption mechanisms could govern the kinetics of distinct adsorption phases. The values of *k*_diff_ and *C* were obtained by linearly graphing *q*_t_ against *t*^1/2^. The plot should be linear and pass through the origin if the rate-controlling step is intraparticle diffusion. The plots in Fig. 9(d) are linear, but they do not pass through the origin. This deviation from the origin or close to saturation may be caused by a change in the mass transfer rate between the start and final stages of adsorption. Additionally, this suggests that there was initial resistance to the boundary layer and that other kinetic models may continuously affect the adsorption rate rather than intraparticle diffusion being the only step that controlled the rate. The correlation coefficient (*R*^2^) and intraparticle diffusion rate constant (*K*_diff_) were obtained from the intraparticle diffusion rate equation (Table 6). Plotting the intra-particle diffusion model against the data with an *R*^2^ larger than 0.9 yields a straight line, as illustrated in Fig. 9(d). This demonstrated that the intra-particle diffusion method was used to adsorb Fe^3+^, Mn^2+^, Ni^2+^, Pb^2+^, NO_3_^−^, PO_4_^3−^, and urea element ions onto the DAP-0.2 Ag NP membrane, as well as the film diffusion or other mechanisms, may be used alone or in conjunction to control the diffusion of element ion adsorption onto the DAP-0.2 Ag NP membrane. These results agree with those reported elsewhere.^64,65^ Three mass transfer processes were included in the adsorption kinetics: the adsorbate was transferred in the liquid film surrounding the adsorbent through external diffusion, also known as film diffusion; the adsorbate was transferred inside the adsorbent's pores through internal diffusion, also known as intraparticle diffusion; and the adsorbate was transferred onto the active sites (Fig. 9(e)).

**Table 6:** Kinetic parameters of Fe^3+^, Mn^2+^, Ni^2+^, Pb^2+^, NO_3_^−^, PO_4_^3−^, and urea element ions onto PVC-DAP-0.2 Ag NP membrane

Metal ions	Kinetic parameters	Fe^3+^	Mn^2+^	Ni^2+^	Pb^2+^	NO_3_^−^	PO_4_^3−^	Urea
1st order	*q*_e,calc_	9.33	7.10	9.02	8.71	6.70	6.47	6.10
	*K*_1,ad_ (g g^−1^ min^−1^)	−0.039	−0.041	−0.036	−0.039	−0.031	−0.036	−0.035
	*R* ^2^	0.966	0.970	0.957	0.968	0.947	0.963	0.971
*q*_e,exp_		11.5	9.9	11	11	10.2	10.4	9.9
2nd order	*q*_e,calc_	11.6	8.6	10.1	10.6	8.7	8.2	7.3
	*K*_2,ad_ (g g^−1^ min^−1^)	0.012	0.016	0.009	0.012	0.013	0.015	0.015
	*R* ^2^	0.989	0.990	0.972	0.986	0.984	0.988	0.985
IPD model	*C*	5.322	3.817	0.934	4.47	3.396	3.536	2.816
	*K*_diff_	0.566	0.435	0.640	0.554	0.469	0.42	0.407
	*R* ^2^	0.935	0.916	0.934	0.903	0.975	0.955	0.944

#### 3.3.7. Surface structure and EDX analysis of the PVC-DAP-0.2 Ag NP membrane before and after element ion adsorption

**Fig. 10:**
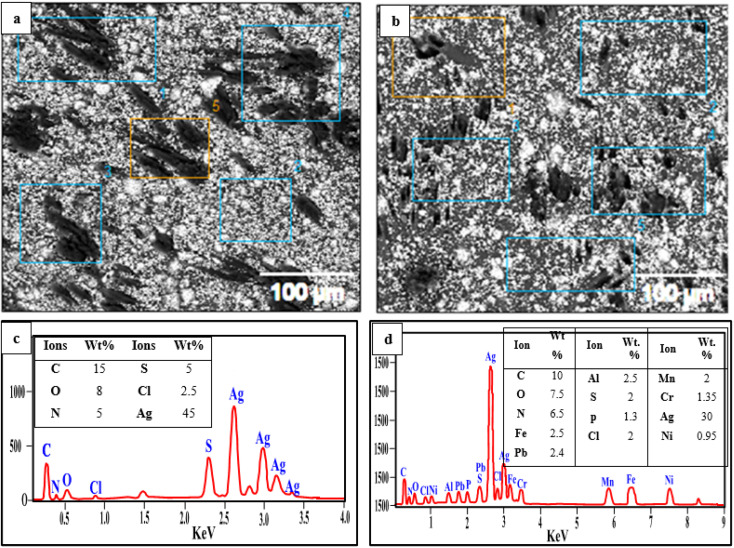
SEM/EDX analysis before (a and b) and after (c and d) the adsorption of Fe^3+^, Mn^2+^, Ni^2+^, Pb^2+^, NO_3_^−^, PO_4_^3−^, and urea element ions by the PVC-DAP-0.2 Ag NP membrane.

The SEM/EDX analyses for the PVC-DAP-0.2 Ag NP membrane before and after element ion adsorption are displayed in Fig. 10(a)–(d). EDX is typically used to examine the molecular structure of solid materials. SEM is a practical method for evaluating the compatibility of various components inside polymeric materials. Using such an approach, the polymeric matrix's numerous interfaces and separation phases, which represent both mechanical and thermal stability features as well as ionic conductivity, can be found. Fig. 10(a) depicts the morphologies of the PVC-DAP-0.2 Ag NP membrane before the adsorption of the selective element. Fig. 10(a) shows the construction of an ice-rock-like configuration. The SEM image in Fig. 10(a) demonstrates how the polymer sample's surface exhibits irregular small-size particles, indicating a high surface area and porous nature. Any adsorbent's large surface area allowed maximum adsorption.^66^ Before adsorption, the SEM images of the PVC-DAP-0.2 Ag NP membrane were distinguished by its regular surface, which had pores of varying sizes and shapes, and a uniform, homogenous shape. The morphological, physical, and molecular structures of the PVC-DAP-0.2 Ag NP membrane have undergone several changes as a result of the uptake and accumulation of element ions. The SEM images of the PVC-DAP-0.2 Ag NP membrane after adsorption showed noticeable modifications in its morphology, including clear deformation, indentations in the PVC-DAP-0.2 Ag NP membrane, denser, packed tightly, and clear and irregular pores. Fig. 10(c) illustrates a characterization of the EDX analysis of the PVC-DAP-0.2 Ag NP membrane before the adsorption of Fe^3+^, Mn^2+^, Ni^2+^, Pb^2+^, NO_3_^−^, PO_4_^3−^, and urea element ions, revealing the existence of C, O, N, Ag, S, and Cl as the main components. Fig. 10(d) displays the appearance of new peaks to confirm the adsorption of Fe^3+^, Mn^2+^, Ni^2+^, and Pb^2+^ ions, verifying that it was adsorbed successfully by the PVC-DAP-0.2 Ag NP membrane. Fig. 10(d) illustrates that a small peak of phosphorus was present, showing that it was adsorbed effectively by the PVC-DAP-0.2 Ag NP membrane, and the peaks of nitrogen increased, confirming the effective adsorption of NO_3_^−^ and urea.

#### 3.3.8. Regeneration studies and reusability

**Fig. 11:**
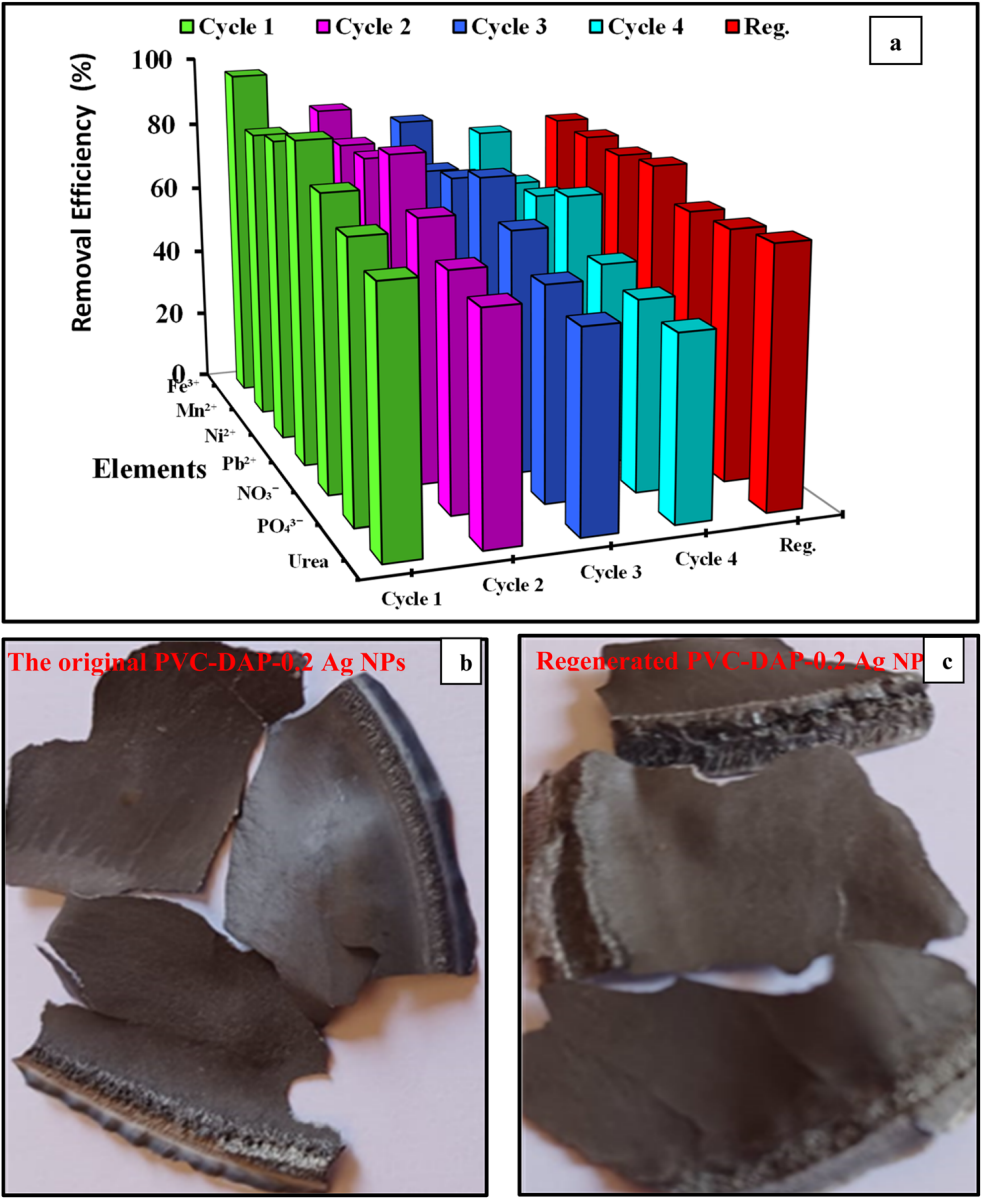
(a) Adsorption capacities of PVC-DAP-0.2 Ag NP membrane after repeated adsorption–desorption operations of Fe^3+^, Mn^2+^, Ni^2+^, Pb^2+^, NO_3_^−^, PO_4_^3−^, and urea ions, metal ion concentration 10 mg L^−1^, pH = 5–7.2 according to the type of metal ions, time starting from 120 min, the adsorbent dose was 3 g L^−1^ and at 35 °C; (b and c) digital images of original PVC-DAP-0.2 Ag NPs and regenerated membrane.

The evaluation of the adsorption material's economical and environmentally beneficial qualities requires regeneration. Desorption and reproducibility in the adsorption process are crucial factors for creating novel adsorbents with practical applications. An important phase in the treatment of contaminated water is the regeneration of the adsorbents employed to extract metal ions.^39^ Because the PVC-DAP-0.2 Ag NP membrane could be employed in all four cycles without changing appearance, the regeneration investigation demonstrated remarkable mechanical stability. The amount of adsorption somewhat decreased after each cycle. Fig. 11(a) shows the effectiveness of the PVC-DAP-0.2 Ag NP membrane in removing the investigated element ions across several cycles (four cycles). It was found that the PVC-DAP-0.2 Ag NP membrane retained nearly 77.4, 76.5, 75.6, 77, 69.2, 69.9, and 71.8% for Fe^3+^, Mn^2+^, Ni^2+^, Pb^2+^, NO_3_^−^, PO_4_^3−^, and urea, respectively, of their initial adsorption capacity after four succeeding adsorption/desorption cycles, which confirmed their effective recoverability. The greatest percentage of element ion adsorption was determined to be in the following sequence order: Fe^3+^ > Pb^2+^ > Ni^2+^ > Mn^2+^ > NO_3_^−^ > PO_4_^3−^ > urea. The testing revealed that the regeneration efficacy (%) of the PVC-DAP-0.2 Ag NP membrane after four repeating cycles was about 77.4, 76.5, 76, 77.1, 69.2, and 71.8%, respectively, for the ions of Fe^3+^, Mn^2+^, Ni^2+^, Pb^2+^, NO_3_^−^, PO_4_^3−^, and urea. Furthermore, it was shown that as the number of regeneration cycles increased, the rate of adsorption was reduced. The reduction in adsorption can be attributed to the incomplete desorption of heavy metals during the desorption process. Besides, the adsorption materials were treated with acid/base to achieve desorption for subsequent regeneration.^8^ This treatment may cause a breakage of PVC-DAP-0.2 Ag NP membrane chains, partly resulting in a reduction in adsorption sites. The PVC-DAP-0.2 Ag NP membrane's capacity to regenerate itself reduced operating costs and demonstrated its industrial applicability. Consequently, the PVC-DAP-0.2 Ag NP membrane was an effective reusable adsorbent that could be used to recover heavy metal ions as well as NO_3_^−^, PO_4_^3−^, and urea from water and wastewater. Fig. 11(b) and (c) show the digital images of the original and regenerated PVC-DAP-0.2 Ag NP membrane, which displays the stability of the membrane after four cycles of reuse. The produced adsorption PVC-DAP-0.2 Ag NP membrane's stable structure and safe adsorption capacity were shown to be present by the findings. The suggested materials continued to regenerate satisfactorily, making them interesting candidates for use in the removal of Fe^3+^, Mn^2+^, Ni^2+^, Pb^2+^, NO_3_^−^, PO_4_^3−^, and urea from contaminated water.

#### 3.3.9. Contaminated groundwater remediation

For humans and all other living things, water is the source of life. The New Valley is one of Egypt's largest governorates, accounting for roughly 44.0% of the country's total land area and 56% of its western desert regions.^67^ Only subsurface water, which originates from the Nubian Sandstone Aquifer, is the main water supply in this region. As a result of the Nile Valley region's inadequate rainfall, underground water in this region has emerged as the primary and possibly the only source for domestic and agricultural uses.^68^ The sediments contained several contaminants that were hazardous to aquatic ecosystems, and anthropogenic activities may significantly affect the creation of mineral pollutants in underground water, consequently changing their chemical compositions. Most water resources in the New Valley region have been polluted with various contaminants, which are extremely dangerous for the health of people, animals, soil, and plants. The seepage of wastewater and the leaching of nitrate and phosphate salts from agricultural fertilization were the main sources of nitrates and phosphate in groundwater in the selected areas. It is important to note that the aquifers comprising Nubian sandstone have a strong propensity for precipitating iron salts.^69^ Therefore, drinking this water may result in difficulties with taste, odor, and stains on clothing. Most sites in the New Valley region have Fe^3^ and Mn^2+^ concentrations above the FAO-recommended upper limit for irrigation water. Additionally, according to WHO (2011) guidelines, Al^3+^, Fe^3^, Mn^2+^, Ni^2+^Cr^2+^, and Pb^2+^ demonstrated potential health risks to humans. According to earlier research, the main exposure method to potentially hazardous elements is by ingesting food and drinking water.^70^ These metals can pose significant risks to human health because they are exposed to people through various mechanisms, including ingestion, oral absorption, and cutaneous absorption.^71^ Therefore, it is important to assess the dangers associated with using these subsurface waters for drinking and irrigation. To prevent potential persistent health risks for both children and adults, purifying water before use should be considered in many regions of the New Valley.

The results of the analyses supported the application of the PVC-DAP-0.2 Ag NP membrane for purifying excess Fe^3+^, Mn^2+^, Ni^2+^, Pb^2+^, NO_3_^−^, PO_4_^3−^, and urea ions from the area's polluted water resources in New Valley. The removal efficiency ranged from 12 to 98% using PVC-DAP-0.2 Ag NP membrane (Table 7). The rejection percentages of the TDS, Ca^2+^, Mg^2+^, Na^+^, K^+^, HCO_3_^−^, SO_4_^2−^, Cl^−^, PO_4_^3−^, NO_3_^−^, Al^3+^, Cr^2+^, Fe^3+^, Mn^2+^, Ni^2+^, Pb^2+^, Cd^2+^, Co^2+^, Cu^2+^, and Sr^2+^ were 46.19, 22.92, 31.43, 59.05, 35.29, 52.29, 52.76, 52.22, 34.96, 81.42, 53.9, 74.1, 85.29, 96.1, 92.7, 98.4, 89, 73.5, 60, 97.5, 45.4, and 68.4, respectively.

**Table 7:** Chemical analyses of major, minor, and trace elements in the contaminated groundwater sample before and after treatment using the PVC-DAP-0.2 Ag NP membrane

Analytical parameter (mg L^−1^)	Before treatment	After treatment	Removed amount (%)
TDS	931	501	46.19
pH	3.70	5.20	—
Ca^2+^	50.6	39	22.92
Mg^2+^	35	24	31.43
Na^+^	210	86	59.05
K^+^	34	22	35.29
CO_3_^2-^	0	0	—
HCO_3_^−^	127	60	52.76
SO_4_^2-^	293	140	52.22
Cl^−^	246	160	34.96
PO_4_^3-^	14.8	2.75	81.42
NO_3_^−^	26.9	12.4	53.90
Al^3+^	3.871	0.983	74.61
Cr^2+^	0.0136	0.002	85.29
Fe^3+^	77.05	2.996	96.11
Mn^2+^	4.059	0.295	92.73
Ni^2+^	0.2537	0.004	98.42
Pb^2+^	0.4553	0.0501	89.00
Cd^2+^	0.0467	0.0124	73.45
Co^2+^	0.1346	0.0537	60.10
Cu^2+^	0.1572	0.004	97.46
Sr^2+^	0.3415	0.1864	45.42
Zn^2+^	1.067	0.3375	68.4

The reduction in the concentricity of major, minor, and trace elements was subsequently a large decline in the salinity of the treated groundwater samples, which reached 46%. The PVC-DAP-0.2 Ag NP membrane created can therefore be advised for use in treatment and desalination processes. One of the key environmental pollutants that had an impact on how various body organs function was heavy metals. The presence of heavy metals in drinking water (surface, ground, and ocean) is a concern for human health and can cause both cancer and non-cancer ailments.^5^ The kidney is the first organ affected by heavy metal poisoning due to its capacity to reabsorb and accumulate divalent metals. The primary kidney damaging heavy metals can result in tubular damage and glomerulopathies are Cr^2+^, Cd^2+^, Pb^2+^, Hg^2+^, Cu^2+^, U^2+^, As^+3^, and Bi^3+^.^72^ It is observed that most residents of the New Valley suffer from some health problems as a result of the high levels of heavy elements, especially Al^3+^, Fe^3+^, Cr^2+^, Mn^2+^, Ni^2+^, and Pb^2+^, in the groundwater used, whether for drinking or agriculture, causing some chronic diseases, especially kidney failure. The study with PVC-DAP-0.2 Ag NP membrane proved that they were able to get rid of heavy and minor elements from the wastewater in the New Valley region, whether ground or surface, which may greatly affect the kidneys, heart and brain. Therefore, it is recommended that these membranes be used as filters for patients with renal failure because they have proven to be highly efficient in removing toxic metals, as well as phosphate, nitrate groups, and urea.

### 3.4. Antibacterial assay

**Fig. 12:**
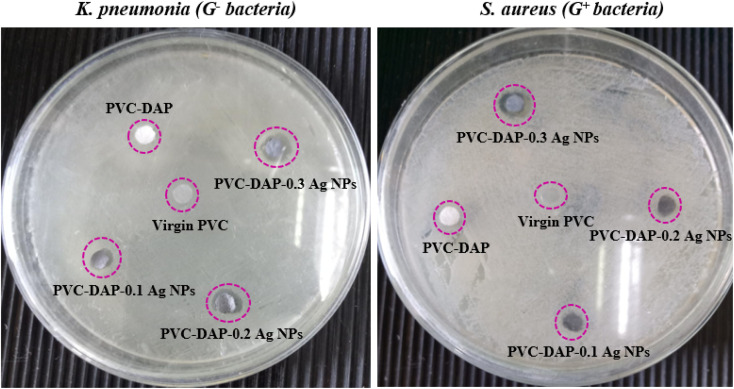
Antibacterial assay of casted nanomembranes against *K. pneumonia* and *S. aureus* bacteria.

The antibacterial test for virgin PVC, PVC-DAP and their nanocomposites with varying proportions of Ag NPs was carried out to assess these materials utilized as nanomembranes for water remediation or biomedical purposes, such as dialysis devices.^73^ This assay was performed against two severe bacteria, such as *K. pneumonia* (*i.e.*, G^−^ bacteria) and *S. aureus* (*i.e.*, G^+^ bacteria). The obtained findings reported in Fig. 12 revealed that virgin PVC and its hybrid negatively affected the growth of selected bacteria in the experiment. Besides, their inhibition zones in the Petri dish were almost zero for the selected bacteria although DAP itself has biological activity for acne treatment as verified elsewhere.^74^ In contrast, halos of inhibition were noticed for nanomembranes with different diameters ranging from ∼6.50 to 14 mm depending on the bacterial type and the Ag NP ratio in the matrix, as shown in Fig. 12 and Table 8. This enhancement was due to the presence of Ag NPs, which were effectively active in hindering the growth of a wide range of G^−^ and G^+^ bacteria by interrupting bacterial developments.^8,29,75^

**Table 8:** Antibacterial activity of virgin PVC, PVC-DAP, and the nanomembranes against two bacteria

	Diameter of the inhibitory zone (mm)	
Composite name	*K. pneumonia* (G^−^ bacteria)	*S. aureus* (G^+^ bacteria)
Virgin PVC	0	0
PVC-DAP	0	0
PVC-DAP-0.1 Ag NPs	7.30 ± 0.20	6.50 ± 0.18
PVC-DAP-0.2 Ag NPs	11.50 ± 0.15	8.20 ± 0.25
PVC-DAP-0.3 Ag NPs	13.00 ± 0.17	14.00 ± 0.21

## 4. Conclusions

In the current context, highly efficient adsorbent nanomembranes were successfully fabricated from Ag NP-doped hybrid PVC-DAP polymer by an *in situ* route to capture many metal ions and contaminates from various water resources. The chemical structures of hybrid PVA-DAP and its nanomembranes were characterized by FT-IR and XRD analyses. The findings demonstrated that the hybridization process of the PVC matrix by DAP was achieved. Further, the data obtained from XRD pattern-based nanomembranes illustrated that the peak intensities increased as the proportion of Ag NPs increased, confirming the embedment of Ag NPs inside the PVC-DAP matrix. A good improvement in tensile and antibacterial properties was observed for the hybrid nanocomposites when Ag NPs were used compared to the PVC-DAP matrix. For adsorption experiments, various element ions, including Fe^3+^, Mn^2+^, Ni^2+^, Pb^2+^, NO_3_^−^, PO_4_^3−^, and urea, were verified. The obtained findings revealed that the optimum nanomembrane was achieved at 0.2% Ag NPs and its percentage of removal effectiveness ranged from 71 to 95%, depending on the metal ion type. This was proven by an SEM-EDX investigation. The Temkin and Freundlich models can describe the adsorption data of the chosen pollutant on the PVC-DAP-0.2 Ag NP membrane in the examined concentration range. The pseudo-2nd-order model fitted the experimental data more accurately than the pseudo-1st-order model, suggesting that a chemical adsorption process might occur. The regeneration efficacy (%) of the PVC-DAP-0.2 Ag NP membrane after four repeating cycles ranged from 69.2 to 77.4%. The endothermic nature of the adsorption process and the increase in randomness at the solid–liquid interface during the adsorption operation were suggested by the positive values of Δ*H*° and Δ*S*° for the Fe^3+^, Mn^2+^, Ni^2+^, and Pb^2+^ ions and *vice versa* in the case of NO_3_^−^, PO_4_^3−^, and urea. The study with the PVC-DAP-0.2 Ag NP membrane proved that they were able to get rid of heavy and minor elements from the wastewater in the New Valley region, whether ground or surface, which may greatly affect human organs, including kidneys. This work depicts a new utilization of these nanomembranes in promising applications, such as wastewater purification and medical sectors.

## Conflicts of interest

The authors have no conflict of interest to declare in this study.

## Supplementary Material

RA-014-D4RA03810J-s001
