# Supplementary material for: Improvement of hybrid polyvinyl chloride/dapsone membrane using synthesized silver nanoparticles for the efficient removal of heavy metals, microorganisms, and phosphate and nitrate compounds from polluted water
Source: RSC Adv. 2024 Jun 18;14(28):19680–700. doi: 10.1039/d4ra03810j (PMC11185225; doi:10.1039/d4ra03810j)
Supplement: RA-014-D4RA03810J-s001 [file RA-014-D4RA03810J-s001.pdf]

Electronic Supplementary Material (ESI) for RSC Advances.
This journal is © The Royal Society of Chemistry 2024

Improvement of hybrid polyvinyl chloride/dapsone membrane using synthesized silver nanoparticles for efficient removal of heavy metals, microorganisms, phosphate, and nitrate compounds from polluted water

Hesham Moustafa[1,2]*, Mohamed A. Shemis[3], Emad M Ahmed[4], Heba Isawi[5]

[1] Polymer Metrology & Technology Department, National Institute of Standards (NIS), Tersa Street, El Haram, P.O Box 136, Giza 12211, Giza, Egypt

[2] Bioanalysis Laboratory, National Institute of Standards (NIS), Tersa Street, El Haram, P.O Box 136, Giza, 12211, Egypt

[3] Department of Biochemistry and Molecular Biology, Theodore Bilharz Research Institute, Giza, Egypt

[4] Department of Physics, College of Science, Taif University, Taif, Saudi Arabia

[5] Water Treatment and Desalination Unit, Hydrogeochemistry Dept., Water Resources and Desert Soils Division, Desert Research Center, P.O.B. 11753, Cairo, Egypt

Table S1: Adsorption isotherms, kinetic models, and adsorption thermodynamics

**(a) Langmuir isotherm**	$$\frac{C_e}{q_e} = \frac{1}{q_m * k_l} + \frac{C_e}{q_m} \qquad (1)$$ Where, q_e (mg/g) is the equilibrium adsorption capacity, C_e (mg/L) is the equilibrium concentration of elemental ions, q_m (mg/g) is the equilibrium maximum adsorption capacity, and K_L (L/mg) is the equilibrium Langmuir constant. The values of k_L and q_m can be calculated using the intercept and slope of the relationship between C_e/q_e and Ce.<br>The non-dimensional equilibrium parameters (R_L) can explain the favorability of this isotherm. The value of RL indicates whether the adsorption environment is linear (RL = 1), favourable (0< R_L < 1), unfavourable (RL > 1), or irreversible (RL = 0). $$R_L = \frac{1}{1 + bC_0} \qquad (2)$$ Where, RL = equilibrium parameter, b (L/mg) is the Langmuir constant, C_o (mg/L) is the initial element ion concentration. The separation factor (R_L) is a non-dimension factor used to anticipate the nature of the procedure and nature of the diagram (Ihsanullah et al., 2020). The R_L value reveals the adsorption environment to be either unfavorable (R_L > 1), linear (R_L = 1), favorable (0< R_L < 1), or irreversible (R_L = 0)
**(b) Freundlich isotherm**	$$log q_e = log K_f + (1/n) \, log \, C_e \qquad (3)$$ Where, n is the adsorption intensity, and k_f is the Freundlich constant, which indicates the adsorbent's capacity for adsorption. The heterogeneity that the Freundlich isotherm depends on is described by the ratio 1/n. A more heterogeneous material is indicated by a smaller value for 1/n.
**(c) Temkin**	$$q_e = B\left(ln\, A + ln\, C_e\right) \qquad (4)$$ Where q_e (mg/g) = the equilibrium amount of adsorbate adsorbed and Ce (mg/L) is the equilibrium concentration of adsorbate. The formula B = RT/b describes B, a constant that relates to the heat of adsorption. The b (J/mol) =

	Temkin constant, R (8.314 J/mol K) = the gas constant, and A (L/g) = the Temkin isotherm constant, all relate to the absolute temperature denoted by T. A and B can be determined from the intercepts (B ln A) and slopes (B) of the qe against ln Ce plot.
**(d) Dubinin-Radushkevich (D-R)**	$lnq_e = lnq_o - \beta\, \varepsilon^2$ (5)<br>$\varepsilon = RTln(1 + \frac{1}{Ce})$ (6)<br>Where q_o = the maximal adsorption capacity, q_e = the quantity of element ions adsorbed per unit weight of the adsorbent (mg/g), β = the activity coefficient helpful in determining the mean sorption energy E (kJ/mol), and Ɛ = the Polanyi potential.<br>$E = \sqrt{1/2\beta}$ (7)<br>Where T is the temperature (K) and R is the gas constant (J/mol K). Calculating q_o and β (mol^2/kJ^2) can affordable from the intercept and slope of the lnq_e against $Ɛ^2$ plot.
**(e) Pseudo-1st-order kinetic**	$Log\,(q_e - q_t) = logq_e - k_1 t$ (8)<br>Where, k1 = pseudo-1st-order rate constant, qe = adsorption capacity at equilibrium (mg/g), qt = adsorption capacity at time t (mg/g), and t = time (minutes). The intercept and slope of log (q_e-q_t) vs t are used to estimate the q_e and k1.
**(f) Pseudo-2nd order kinetic**	$\frac{t}{q_t} = \frac{1}{k_2\, q_e^2} + \frac{t}{q_e}$ (9)<br>Where, k_2 = pseudo-2nd-order constant, and q_e = adsorption capacity at equilibrium (mg/g) and q_t = adsorption capacity at time t (mg/g), t = time (minutes). The slope and intercept of t/q_t plotted against t are used to determine the values for q_e and k_2.
**(g) Intraparticle diffusion rate**	$q_t = K_{diff}\, t^{½} + C$ (10)<br>Where, q_t (mg/g) is the amount of adsorbed (heavy metal/element) at time t; K_{diff} (mg/g·min−0.5) is the rate constant; $t^{½}$ is the square root of contact time

	(min$^{0.5}$); and C is the intercept.
**(h) Adsorption thermodynamics**	$$\ln K_c = (-\Delta H/R).(1/T) + (\Delta S/R) \quad (11)$$ Where, ΔH= enthalpy of the system (how much energy is released or absorbed) and ΔS = entropy (measure of randomness in the system). $$\Delta G = -RTlnK_c \qquad (12)$$ Where, ΔG = change in Gibbs free energy, R = universal gas constant (8.314 J/mol..K), T = temperature (°K), and K_c = thermodynamic equilibrium constant (q_e/C_e), q_e (mg/L) is the adsorbed element (initial-final concentricity), C_e (mg/L) is the residual (final concentricity).

3.1.2. Effect of pH

The degree of ionizable metal ions in their defined chemical state is quickly influenced by pH, which is one of the essential factors that determines the efficacy of the sorption process [1]. In addition to affecting the surface charge of the adsorbent and the state of the adsorbate in solution, the pH of the solution has a significant impact on the adsorption process. Fig. S1 illustrates the experimental results on the influence of pH on the % removal of Fe^{3+}, Mn^{2+}, Ni^{2+}, Pb^{2+}, NO_3^-, PO_4^{3-}, and urea from binary solution by PVC-DAP-0.2 Ag NPs membrane, Fig. S1. The amount of element ions that are absorbed up increases as the pH value rises, with maximal adsorption occurring at pH values of 5 for Fe^{3+}, Mn^{2+}, Ni^{2+}, NO_3^- and PO_4^{3-}, and 7.2 for Pb^{2+} and urea and reduced at inferior or greater pH values. The data display that for PVC-DAP-0.2 Ag NPs membrane with increasing pH from 3 to 5 the element ions Fe^{3+}, Mn^{2+}, Ni^{2+}, NO_3^-, and PO_4^{3-} adsorption rate increases to reach the optimum value at pH= 5 and with increasing pH from 5 to 7.2, the Fe^{3+}, Mn^{2+}, Ni^{2+}, NO_3^-, and PO_4^{3-} adsorption is reduced gradually. These behaviors are responsible for the protonation and deprotonation of the reactive groups of the PVC-DAP-0.2 Ag NPs membrane as well as, the change in the ionic conditions of the functional sulfonyl (O=S=O) and amine (NH_2) groups. A lot of the amino groups in dapsone are easily protonated and produce positively charged groups in acidic conditions that are responsible for the electrostatic repulsion between the metal ions positively charged and positively charged surface of PVC-DAP-0.2 Ag NPs membrane. In addition to electrostatic repulsion, the increased competitive effect and diffusion resistance produced by hydroxide ions could be responsible for the poorer removal efficiency under alkaline conditions. Electrostatic repulsion made it difficult for anions to bind to the negatively charged surface, therefore

additional mechanisms like ion exchange and hydrogen bonds should be used during the sorption process [2].

Due to electrostatic repulsion on the bonding location of the adsorbent site, the removal effectiveness of Fe^{3+}, Cr^{2+}, Mn^{2+}, Ni^{2+}, Pb^{2+}, and urea was low under an acidic state. Due to the high H^+ concentration, metal ions and H^+ ions compete for replaceable cations on the surface of the PVC-DAP-0.2 Ag NPs membrane, which may explain some of the slight sorption at lower pH levels. Higher pH causes the selected metal ions to precipitate as hydroxides ($M(OH)_2$), which reduces removal efficiency and minimizes adsorption values. This lowers the rate of adsorption and, consequently, the effectiveness of metal ion removal.

The optimum PO_4^{3-} removal at pH=5 is consistent with that which has been established previously for the adsorption of PO_4^{3-} using a polyethersulfone-type affinity membrane [3]. Phosphate adsorption rate was determined by the membrane's surface charge as well as the various ionic forms of phosphate ions present at different pH levels, such as $H_2PO_4^-$=2.15, HPO_4^{2-}=7.20, and $PO4^{3-}$=12.33, respectively [4].

It was proposed that the membrane eliminated PO_4^{3-} ions as $H_2PO_4^-$ from the system. As the pH rises above 5, it is hypothesised that the membrane's surface will become positive rather than negative. The amine group's nitrogen atoms are protonated since the pH is between 3.0 and 5.0, which will help with the uptake of PO_4^{3-} ions. However, when the pH is lower than 5.0, some of the $H_2PO_4^-$ anions will transform into H_3PO_4 molecules. Additionally, because the pH is lower than 5, PO_4^{3-} will also exist as H_3PO_4. These facts may contribute to a reduction in the membrane's uptake of PO_4^{3-} ions. Additionally, as pH rises above 5.0, the membrane's deprotonated amine and sulfonyl groups may have less affinity for PO_4^{3-} ions, which will result in less PO_4^{3-} ions adsorption. Above pH 6, HPO_4^{2-}, a phosphate species with a stronger negative charge than $H_2PO_4^-$, becomes the predominant species in the solution. The phosphate adsorption performance can be significantly reduced by the strong molecule repulsive contact that is formed between the negatively charged PVC-DAP-0.2 Ag NPs membrane surface and HPO_4^{2-} as well as by the competition between HPO_4^{2-} and PO_4^{3-} ions. Also, a lot of OH^- ions compete with PO_4^{3-} ions to be adsorbed on the PVC-DAP-0.2 Ag NPs membrane surface, which reduces the rejection of PO_4^{3-} ions by the membrane. The NO_3^- are also negative as in the case of PO_4^{3-}, repulsion forces between ions and adsorbent in alkaline circumstances. It is most likely that the increased competition between OH- ions and nitrate ions for sites is what causes the low efficiency of ion removal in alkaline circumstances.

Based on the pH of the medium, urea can be protonated or deprotonated. The optimum removal efficiency of urea was at pH=7.2 and decrease with lower or higher levels of pH. When the pH is lower than 7.2 the protonation of the amino groups present in urea will occur. At lower pH, the amino groups in urea are present in their ionized form (NH_3^+), which are compete with H^+ ions in acidic solution and impeded the NO_3^- adsorption. However, low pH resulted in electrostatic repulsion between urea and the reactive functional groups in the PVC-DAP-0.2 Ag NPs membrane surface, leading to lower removal efficiency. When the pH value =7.2, the functional groups in the PVC-DAP-0.2 Ag NPs membrane are expected to be in their ionized and non-ionized form, while the amine groups of urea remain in their non-ionized form (primary amine, NH_2) causing an increase in interaction via hydrogen bonding of the carbonyl groups in urea so the removal efficiency increased.

Regarding NO_3^-, the increase of H^+ concentration at pH < 6 impeded the NO_3^- sorption, and there is an adsorption competition with Cl^- anion from HCl utilized to modify the pH of the solution [5]. At pH > 7.2, the NO_3^- capacity for adsorption also decreased due to the adsorption competition with OH^- [6]. The OH^- ions and nitrate ions compete for the same membrane active sites in a basic solution.

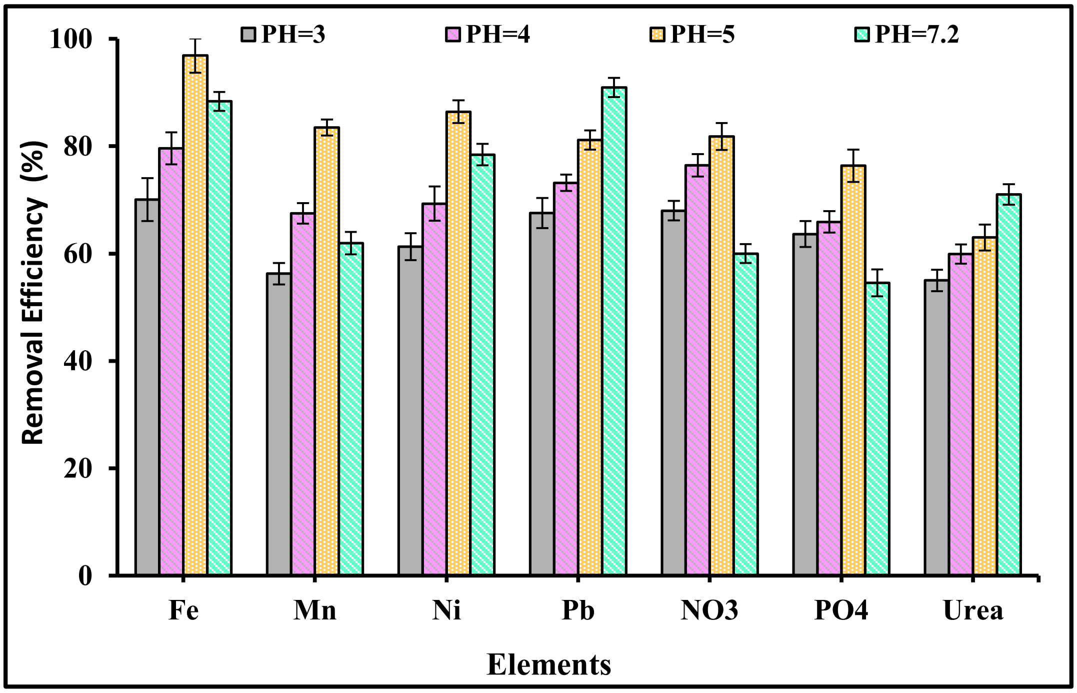

Fig. S1: Effect of pH on adsorption of Fe^{3+}, Mn^{2+}, Ni^{2+}, Pb^{2+}, NO_3^-, PO_4^{3-}, and urea, element ions concentration 10 mg/L, Temp. = 35 °C, pH ranges from 5 to 7.2 according to the type of metal ions, time 120 min., adsorbent dose is 3 g/ L onto PVC-DAP-0.2 Ag NPs membrane.

References

[1] Isawi, H., 2020. Using Zeolite/Polyvinyl alcohol/sodium alginate nanocomposite beads for removal of some heavy metals from wastewater. Arab. J. Chem. 13, 5691–5716. https://doi.org/10.1016/j.arabjc.2020.04.009.
[2] Cui X., Li H., Yao Z., Shen Y., He Z., Yang X., Ng H.Y., Wang C.-H. (2019) Removal of nitrate and phosphate by chitosan composited beads derived from crude oil refinery waste: Sorption and cost-benefit analysis. Journal of Cleaner Production. 207, (2019), 846-856. https://doi.org/10.1016/j.jclepro.2018.10.027.
[3] Wang X, Song L, Yang F, He J (2016) Investigation of phosphate adsorption by a polyethersulfone-type affinity membrane using experimental and DFT methods. Desalin. Water Treatm. 57(52):25036–25056.
[4] Banu H.T., Meenakshi S., One pot synthesis of chitosan grafted quaternized resin for the removal of nitrate and phosphate from aqueous solution, Int. J. Biol. Macromol. 104 (2017) 1517–1527.
[5] Zhang Q., Zhang Z., Teng J. et al., "Highly efficient phosphate sequestration in aqueous solutions using nanomagnesium hydroxide modified polystyrene materials," Industrial & Engineering Chemistry Research, vol. 54, no. 11, pp. 2940– 2949, 2015.
[6] Yan L.-G., Yang K., Shan R.-R., et al., "Kinetic, isotherm and thermodynamic investigations of phosphate adsorption onto core-shell Fe_3O_4@LDHs composites with easy magnetic separation assistance," Journal of Colloid and Interface Science, vol. 448, pp. 508–516, 2015.
